# Optimization and performance prediction of air-flotation cyclones for oily wastewater

**DOI:** 10.1039/d6ra03194c

**Published:** 2026-08-24

**Authors:** Qian Huang, Yuxin Xie, Yufan Yang, Zhe Shen, Ruilin Wang, Xicheng Zhang, Huirong Huang, Yuan Tian, Xueyuan Long

**Affiliations:** a College of Petroleum and Natural Gas Engineering, Chongqing University of Science and Technology Chongqing 401331 China huangqianswpu@163.com 2024201107@cqust.edu.cn 2024201088@cqust.edu.cn 2024201066@cqust.edu.cn hryh@cqust.edu.cn zqianshu770@gmail.com 2007078@cqust.edu.cn xieyuxincqust@163.com; b Sichuan Guangran Pipeline Engineering Co., Ltd China 3218695914@qq.com

## Abstract

Treating heavy oil-containing wastewater on offshore production platforms remains challenging because heavy oil has a density close to that of water and dispersed droplets are difficult to remove using conventional centrifugal separation alone. To address this limitation, this study proposes an integrated treatment process coupling centrifugal separation with dissolved air flotation and develops a modified Bloom–Heindel interaction model for air-flotation cyclones. The proposed model explicitly incorporates the complete bubble-droplet interaction sequence, including collision, attachment, and stabilization-detachment in a high-shear swirling centrifugal field. Compared with a conventional model that neglects bubble-droplet micro-interactions and may produce prediction errors of up to 15%, the modified model reduces the average deviation from field measurements to 1.2%. Based on the validated model, computational fluid dynamics simulations were performed to analyze velocity, pressure, turbulent kinetic energy, and oil-phase concentration distributions. To reduce the computational cost of repeated multiphase simulations, a backpropagation neural network was established to predict separation efficiency under different structural and operating parameters. The independent test results show a Pearson correlation coefficient greater than 0.99, a mean squared error of 0.00223785, and more than 95% of prediction errors within ±5%. Particle swarm optimization was further used to determine the optimal design under field-oriented conditions. The optimized configuration, including a 6.4 mm overflow pipe diameter and a 13° large cone angle, yields a theoretical maximum oil removal efficiency of 95.71%. This coupled physical modeling, CFD simulation, neural-network prediction, and intelligent optimization framework provides a quantitative basis for improving compact oily wastewater treatment equipment on offshore platforms.

## Abstract 2

An integrated CFD and machine learning framework optimizes air-flotation cyclones for offshore heavy oil wastewater, achieving 95.71% theoretical separation efficiency.

## Graphical abstract

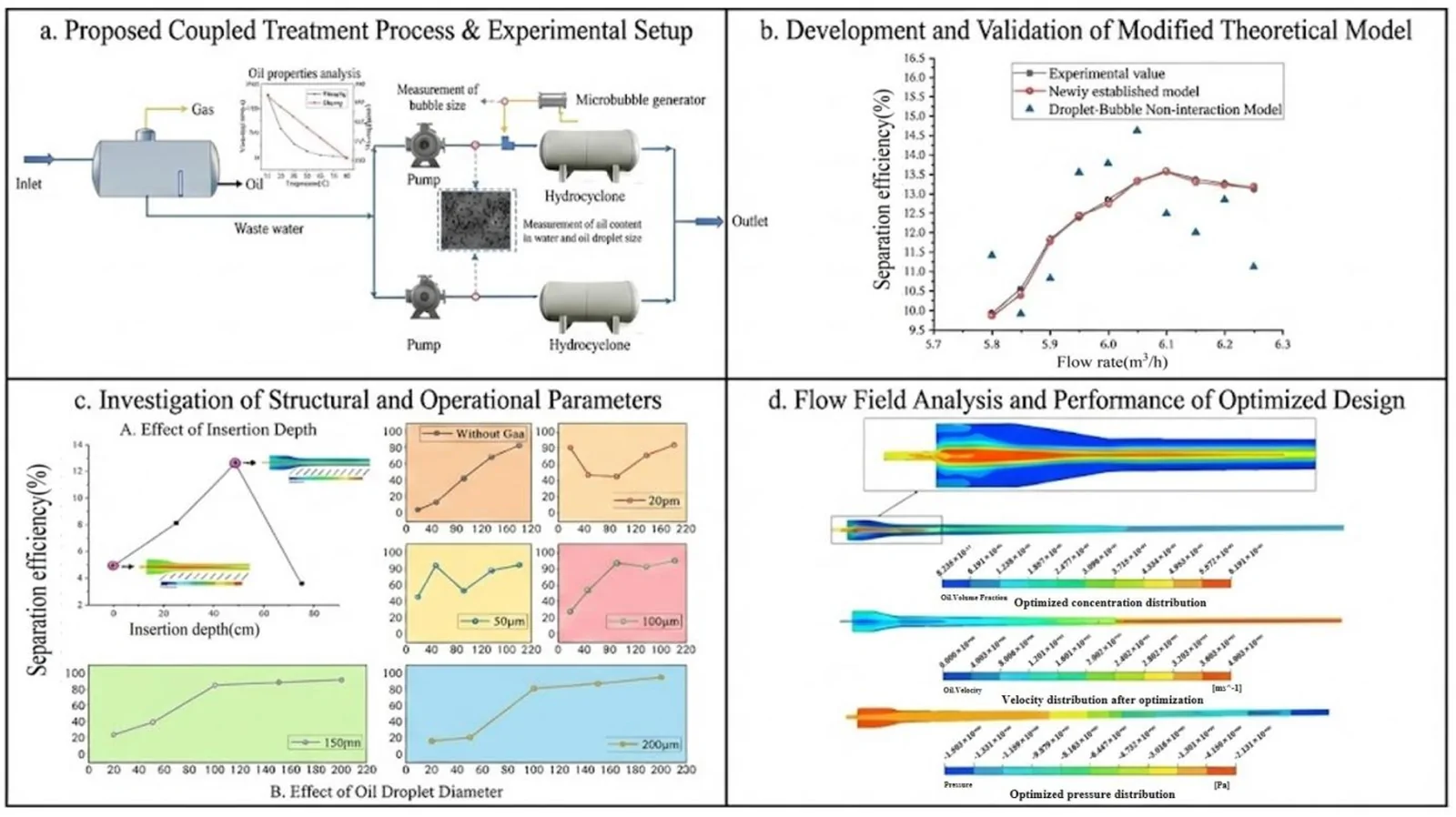

## 1 Introduction

China ranks fourth worldwide in viscous oil resources. More than 70 viscous oil fields have been discovered in China.^1^ Proven onshore viscous oil reserves are approximately 4 billion tons, while proven offshore reserves are about 4.2 billion tons. With increasing attention to marine environmental protection, oily wastewater generated during heavy oil production has become a major constraint on the sustainable development of offshore oilfields.^2,3^ Hydrocyclone separators are widely used on offshore platforms for the treatment of oil-in-water emulsions. However, heavy oil has a density close to that of water. The small density difference weakens the centrifugal driving force for phase separation. As a result, conventional hydrocyclones often show limited removal efficiency for fine oil droplets and poor overall separation performance. To overcome these limitations, coupling centrifugal separation with dissolved air flotation has become a promising strategy for oily wastewater treatment.

Current studies on air-flotation cyclone processes mainly focus on experimental investigation, theoretical modeling, and numerical simulation. Experimental studies can be divided into two main categories. The first category focuses on bubble distribution and motion in cyclone flow fields. Wang *et al.*^4,5^ used visualization devices to compare bottom and side bubble injection modes. They analyzed bubble size distribution and bubble trajectories under different injection conditions. Li *et al.*^6–8^ used camera-based bubble detection techniques to compare different bubble generation devices, including jet and microporous devices. They also discussed bubble coalescence during upward migration in the cyclone field. The second category focuses on the effects of operating parameters on oil-water separation efficiency. Liu^9^ investigated the influence of medium particle size on separation efficiency. The results showed that increasing the oil phase volume fraction and gas volume, or reducing bubble size, could improve air-flotation separation efficiency. Xu *et al.*^10^ performed multi-factor orthogonal experiments to evaluate the effects of feed velocity and gas volume fraction on the performance of an oil-water flotation column. They also determined suitable ranges for key operating variables. Ran *et al.*^11^ studied a cyclone-static micro-bubble flotation column and analyzed the effects of circulating pressure and gas flux. Their results indicated that increasing gas holdup and optimizing bubble size distribution are effective ways to improve flotation performance. Bai *et al.*^12^ developed a cyclone-air flotation synergistic device. When the gas mass ratio ranged from 0.003 to 0.020 and the inlet oil concentration ranged from 50 to 300 mg L^−1^, the separation efficiency increased by 10–50% compared with that of conventional cyclone equipment. Luo *et al.*^13^ developed a cyclone-air flotation evaluation system. They investigated the effects of centrifugal intensity, wastewater properties, and bubble-oil droplet size matching on the flotation process. They also optimized the operating parameters of a low-intensity cyclone-air flotation device. The dynamic behavior of complex multiphase systems has also been investigated. Liu *et al.*^14–19^ developed a two-way coupled discrete-continuous phase model for foam systems containing proppant particles. Based on the Euler–Lagrange framework, they proposed a particle trajectory prediction algorithm. The use of a virtual collision grid improved computational efficiency by more than 40%. Krause and co-workers^20^ developed a phase-field method for tracking three-phase interfaces. By using a modified Cahn–Hilliard equation, the prediction error of oil-water interfacial tension was controlled within 5%. Their study showed that secondary vortices in the cyclone field play an important role in oil film stripping. When the vortex intensity exceeded 35 s^−1^, the wall oil film stripping efficiency reached more than 92%. Kim and co-workers^21^ developed a modified Casson model for high-viscosity crude oil. By introducing a correction term for the strain-rate history effect, they reduced the viscosity prediction error from 18.6% to 7.3%.

Numerical simulation has become an important tool for analyzing multiphase flow in air-flotation cyclone systems. Wang *et al.*^22–24^ constructed a three-dimensional VOF multiphase flow model using ANSYS Fluent. They quantified the optimal ratio between the gas injector outlet diameter and the separation chamber diameter. Li *et al.*^25^ coupled the population balance model with the discrete phase model to reveal the effect of gas injection pressure on bubble size distribution. For polymer-containing produced liquid, an improved SST *k*–*ω* model^26^ was developed to capture the sharp decrease in apparent viscosity when the shear rate exceeded 500 s^−1^. This viscosity reduction increased the radial migration velocity of the oil phase by 35%.^27,28^ Overall, numerical simulation provides an effective method for evaluating the performance of air-flotation cyclones. However, existing models have not fully incorporated the interactions between bubbles and oil droplets, especially collision, attachment, and stabilization.^29^ This limits their ability to describe the separation behavior of heavy oil droplets in high-shear centrifugal fields. To address this gap, this study develops a modified model based on force analysis. The model is used to investigate an improved process that couples cyclone flow characteristics with air-flotation effects. It also provides a basis for analyzing the effects of structural and operating parameters on the separation efficiency of air-flotation cyclones.^31^

Recent studies have also reported advances in alternative oil-water and wastewater separation methods. These include chemically enhanced wastewater treatment, high-pressure dewatering, membrane-based separation, and numerical modeling of compound droplet deformation and breakup.^33–38^ These methods provide useful theoretical and technical references. However, offshore platforms require compact treatment equipment with high capacity, small footprint, and strong adaptability to fluctuating produced-water conditions. Therefore, air-flotation cyclones remain attractive for offshore wastewater treatment. Their further development requires a more accurate description of micro-scale bubble-droplet interactions and macro-scale swirling separation behavior.

Despite previous progress, three scientific gaps remain. First, most flotation and cyclone models simplify bubble-droplet interactions. They do not fully describe collision, attachment, and stabilization-detachment under high-shear centrifugal conditions. Second, the coupling between micro-scale interfacial behavior and macro-scale velocity, pressure, and turbulent kinetic energy distributions inside air-flotation cyclones remains insufficiently quantified. Third, repeated CFD simulations for multi-parameter optimization are computationally expensive. A physically validated data-driven surrogate model is therefore needed for rapid engineering design.

Accordingly, the objectives of this study are fourfold. First, a modified bubble-droplet interaction model is developed to describe collision, attachment, and stability in a swirling centrifugal field. Second, CFD is used to reveal the flow-field characteristics and separation mechanism of the air-flotation cyclone. Third, a BP neural-network surrogate model is established for rapid prediction of oil-water separation efficiency. Finally, particle swarm optimization is used to optimize the key structural and operating parameters and to provide field-oriented guidance for equipment modification.

## 2 Experimental

**Fig. 1:**
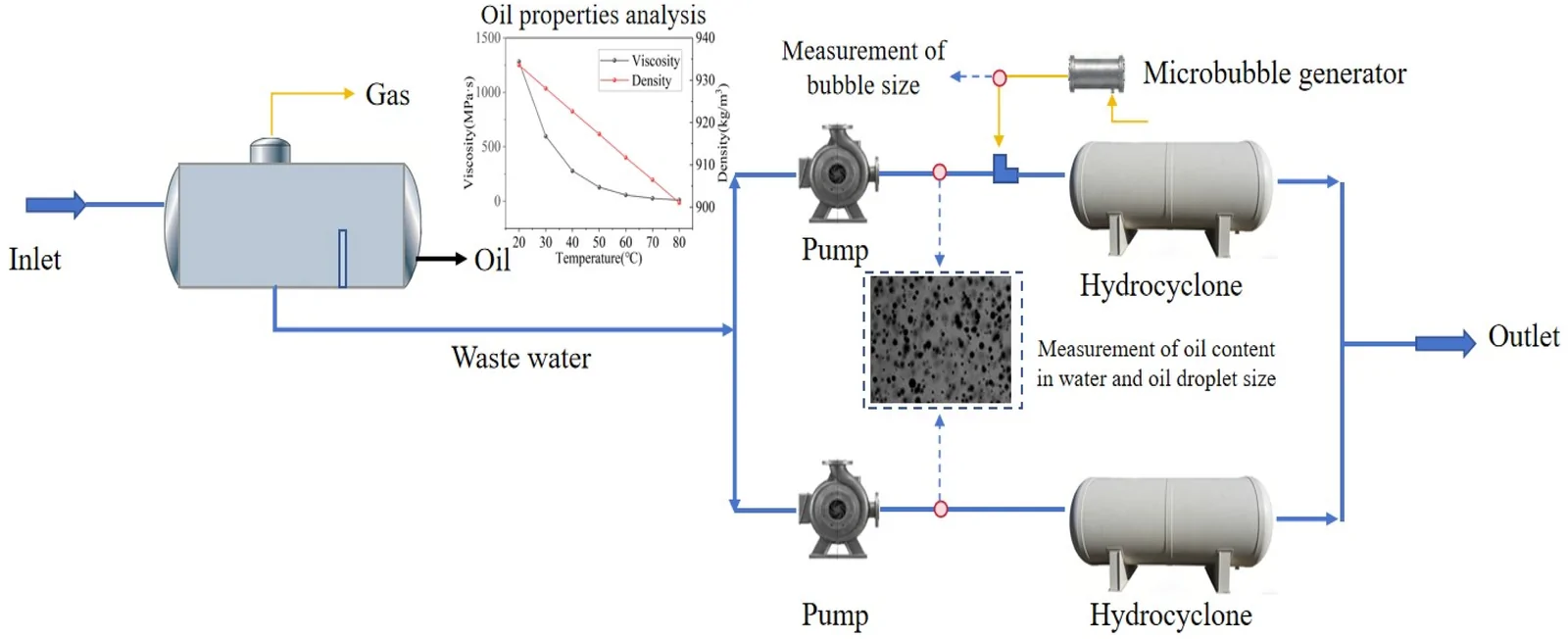
Experimental process flow chart.

**Fig. 2:**
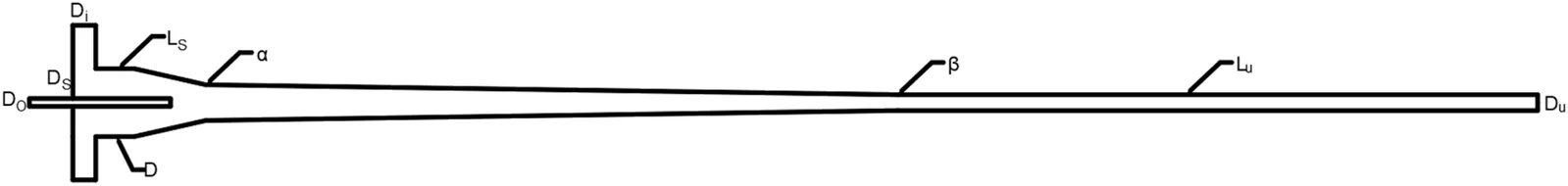
Schematic diagram of hydrocyclone tube structure.

Fig. 1 depicts a complete wastewater treatment system, which is composed of a separator, pumps and hydrocyclone separators. As illustrated in Fig. 1, two hydrocyclones are arranged in parallel. A single hydrocyclone consists of 128 cyclone tubes connected in parallel; the structural configuration of the cyclone tube is shown in Fig. 2 and its structural parameters are presented in Table 1. Separation efficiency of the hydrocyclone is defined as the core evaluation parameter, calculated as the ratio of the mass flow rate of the target component at the overflow outlet to the total mass flow rate of the oil phase at the inlet. The expression is shown in eqn (1).1
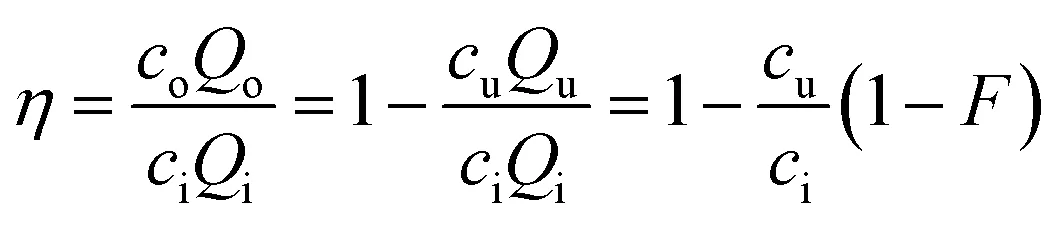
In the equation, *η* represents the separation efficiency of the cyclone; *Q*_u_ is the flow rate at the cyclone's underflow outlet, in kg h^−1^; and *c*_i_, *c*_o_, *c*_u_ are the oil concentration (in mg L^−1^) at the cyclone inlet, overflow outlet, and underflow outlet, respectively.

**Table 1:** Cyclone tube size parameters

Structural parameters	Parameter	Value
*D*_i_ (mm)	Inlet dimensions	16
*D*_s_ (mm)	Large cone section outlet diameter	24.8
*D*_o_ (mm)	Overflow outlet diameter	5.4
*D*_u_ (mm)	Underflow outlet diameter	11
*D* (mm)	Cylindrical section diameter	47
*L*_s_ (mm)	Cylindrical section length	42.5
*L*_u_ (mm)	Tailpipe length	442
*α* (°)	Large cone angle	13
*β* (°)	Small cone angle	1

**Fig. 3:**
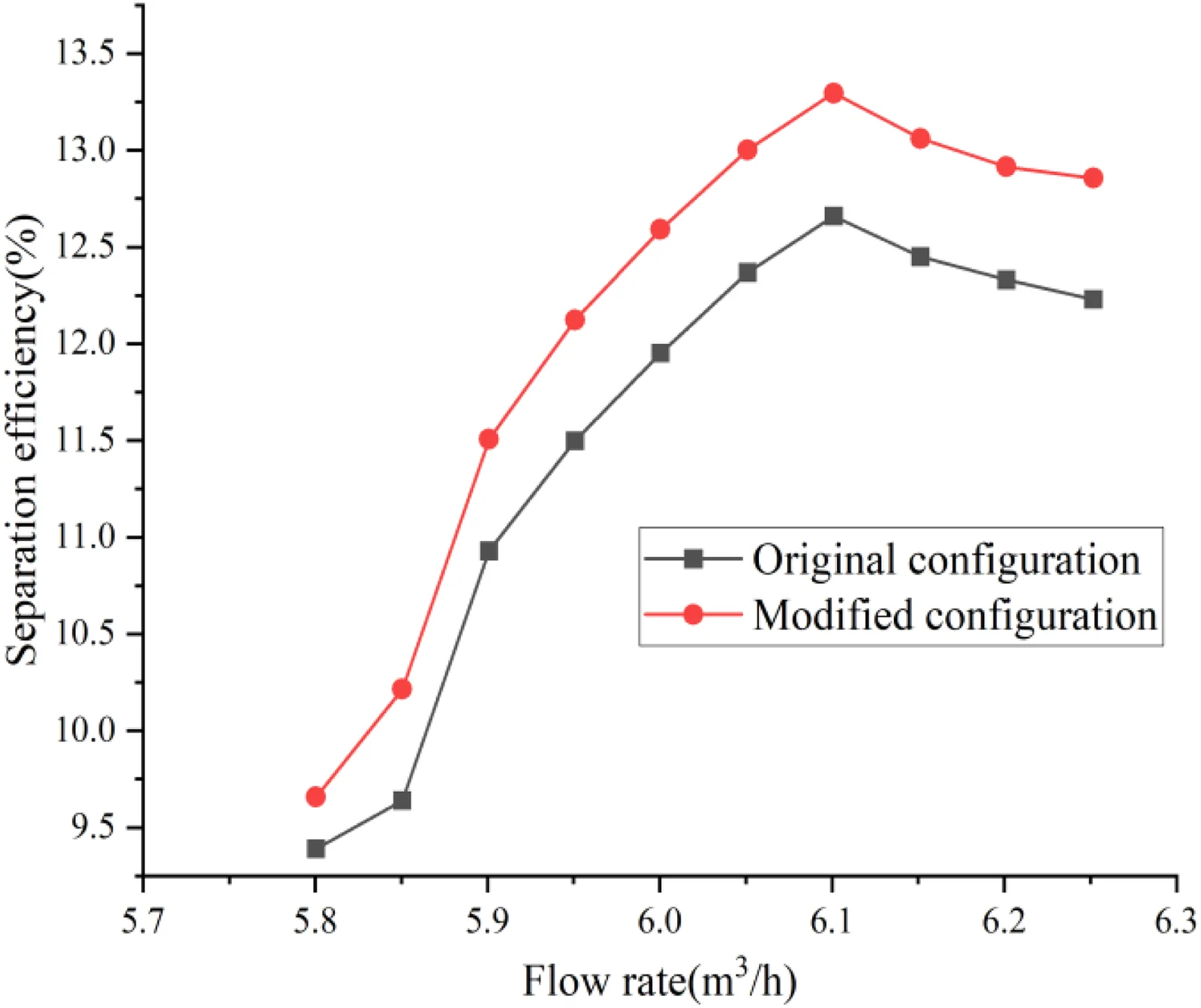
Comparison of separation efficiency before and after modification.

To investigate the impact of cyclone tube flow rates on separation efficiency under constant total processing conditions, oil droplet size characteristics were measured using a digital microscopy system to detect the inlet and outlet water samples. Image processing algorithms were applied to obtain the oil droplet size distribution spectrum. The quantitative analysis of the oil phase concentration was carried out using a WILKS InfraCal CVH infrared oil content analyzer, which complies with EPA 413.2 standards. The extraction process strictly followed the perchloroethylene solvent method, ensuring that the test results met the required specifications. Continuous monitoring over 96 hours revealed that the median droplet size at the inlet remained stable at 50.37 µm, indicating good dynamic stability of the emulsified system. By dynamically adjusting the number of parallel cyclone units and testing the collaborative operation mode of the separators, oil phase residual concentration and droplet size distribution patterns were obtained at different treatment flow rates (5.80–6.25 m^3^ h^−1^) under the average inlet oil concentration of 360 mg L^−1^. Based on the separation efficiency calculation model established in eqn (1), the separation efficiency of a single cyclone tube and its processing capacity were calculated. The relationship between the two was found to be nonlinear, and the inlet flow rate-separation efficiency correlation curve constructed is shown in Fig. 3. The field data in Fig. 3 show that there is a critical threshold effect between the processing capacity and separation efficiency of the cyclone separation system. When the treatment flow rate is below the critical value of 6.1 m^3^ h^−1^, the separation efficiency is positively correlated with the flow rate. However, once the flow rate exceeds this critical threshold, the separation efficiency begins to decrease.

To further improve the oil-water separation efficiency, this study carried out technical upgrading of the existing process. Based on the *in situ* cyclone tubes (see Fig. 2), and under the premise of strictly controlling the transformation cost and minimizing the impact on oilfield production, an aeration system modification scheme was designed to realize the organic coupling of air flotation technology and cyclone technology. Specifically, the microbubbles generated by the bubble generator were injected into the inlet of the hydrocyclone. Based on the modified device, field tests were conducted, with the average bubble diameter generated by the bubble generator controlled at 50 µm. The oil-water separation efficiency obtained from these tests is shown in Fig. 3. After aeration, the oil-water separation efficiency significantly improved, with an increase of 5%. To further enhance the oil-water separation efficiency, numerical simulation methods will be employed in the next step to further optimize the process.

To improve the reproducibility of the field validation, the main measured properties of the heavy oil used in this study are supplemented here. The crude oil had an acid value of 2.10 mg KOH per g, a cloud point of 20.57 °C, a wax content of 12.32 wt%, a resin content of 11.27 wt%, an asphaltene content of 2.51 wt%, and a sulfur content of 0.23 wt%. These composition characteristics indicate that the tested oil was a typical heavy oil system with high interfacial complexity, which helps explain the difficulty of separating small dispersed droplets using conventional centrifugal separation alone.

## 3 Model description

### 3.1 Numerical calculation model

#### 3.1.1 Oil droplet-gas bubble interaction

In the air-flotation cyclone, the collision, adhesion, and detachment processes between oil droplets and bubbles are the core interfacial behaviors that affect oil-water separation efficiency. Considering the limitations of traditional flotation mineral process calculation models in oil-water systems, this study analyzes the impact of the physical property differences between oil droplets and mineral particles on the bubble interaction mechanism based on multiphase flow dynamics and interfacial chemistry theory. Improved models for collision probability, adhesion probability, and stability probability are developed. Additionally, a capture efficiency model coupling multiple physical field effects is constructed based on the principle of mass conservation.

The novelty of the modified model lies in extending the original Bloom–Heindel framework from a relatively quiescent or gravity-dominated flotation environment to a high-shear centrifugal field. In the collision stage, the conventional velocity difference is replaced by a turbulence-induced relative velocity ratio. In the attachment stage, the force balance is expanded to include centripetal buoyancy, fluid drag, Saffman lift, and Magnus force. In the stability stage, the conventional Bond number is reconstructed as a dynamic bond number that incorporates centrifugal shearing and mechanical acceleration. Therefore, the present model is not a simple combination of CFD and machine learning, but a cross-scale physical framework linking micro-scale bubble-droplet interactions with macro-scale air-flotation cyclone flow structures.

The main assumptions used in this model are also clarified. Bubbles and oil droplets are treated as spherical dispersed phases within the dilute operating range considered in this study. Secondary coalescence and breakup are not explicitly solved in the current model. The physical properties of oil and water are assumed to remain constant at the measured operating temperature of 53 °C. These assumptions make the model computationally tractable while maintaining sufficient accuracy for the field-oriented conditions investigated here. Their limitations are further discussed in the engineering applicability and future work sections.

The capture efficiency of a particle and a bubble can be defined as follows:^27^

2 *p*_t_ = *p*_c_*p*_a_*p*_s_

where *p*_c_ is the collision efficiency, *p*_a_ is the attachment efficiency, and *p*_s_ is the stability efficiency.

##### 3.1.1.1 Collision of oil droplets and bubbles

The collision probability can be defined as the likelihood that a mineral particle/oil droplet, when passing by a bubble, will form a thin water film as it gradually approaches the surface of the bubble. In the development of the collision model for oil droplets and bubbles, the collision probability caused by gravitational and inertial effects can be considered negligible (*i.e.*, 0). The collision mechanism between oil droplets and bubbles involves interception effects and turbulent interactions. The collision efficiency can be calculated using eqn (3).

3 *p*_c_ = 1 − (1 − *p*_ci_)(1 − *p*_ct_)

In the equation, *p*_c_ represents the total collision probability, *p*_ci_ is the collision probability caused by the interception effect, and *p*_ct_ is the collision efficiency resulting from turbulent interactions.

Based on Newton's second law, the force balance relationship between oil droplets and bubbles can be established, as shown in eqn (4).4
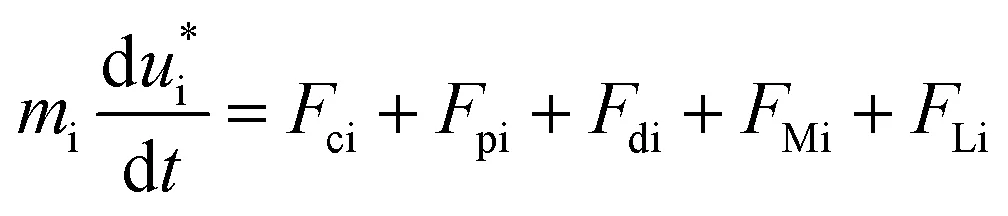
In the equation, *i* = *p* represents the oil droplet, and *i* = *b* represents the bubble. *F*_c_, *F*_p_, *F*_d_, *F*_M_, *F*_L_ correspond to the following forces: *F*_c_ is the inertial centrifugal force, *F*_p_ is the centripetal buoyant force, *F*_d_ is the fluid medium resistance, *F*_M_ is the Magnus force, and *F*_L_ is the Saffman force. The detailed expressions of these force terms are provided in Appendix A.

By combining the definitions of the forces, the corrected velocities of the oil droplet 
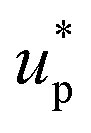
 and the bubble 
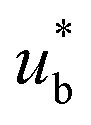
 can be obtained. Then, using eqn (10), the corrected velocity ratio can be calculated.5
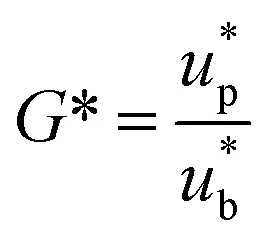


Based on the throttling effect, the collision probability between the bubble and oil droplet obtained from the Heindel–Bloom model^30^ is as follows:6
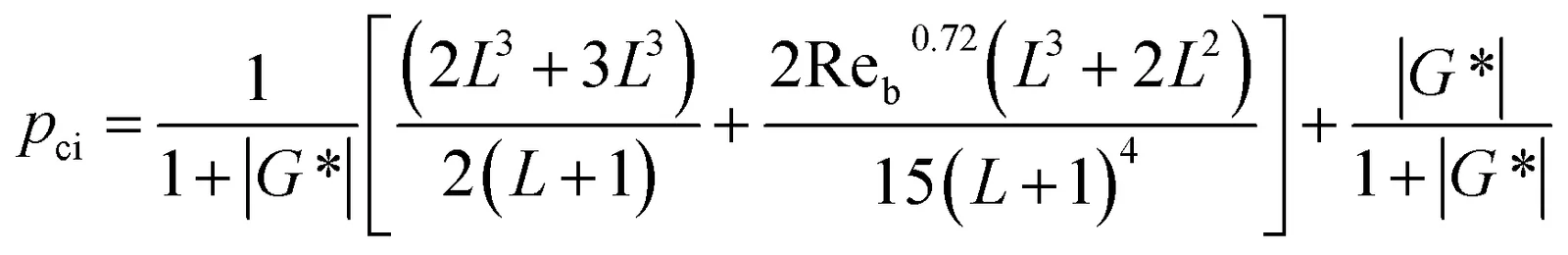


Additionally, the collision between oil droplets and bubbles is also influenced by turbulent effects, which were often neglected in previous collision probability calculations. In Kostoglou's study^30^ on the collision behavior of oil droplets and bubbles in a turbulent flow, the bubble velocity in the Yoon–Luttrell collision probability model was replaced with the relative velocity between oil droplets and bubbles caused by fluid pulsations, thus enabling the calculation of collision probability between oil droplets and bubbles in a turbulent field.

In this study, following Kostoglou's approach of modifying the Yoon–Luttrell model, this method is extended to the Heindel–Bloom model. As a result, an expression for the collision probability between oil droplets and bubbles caused by turbulence is derived, as shown in eqn (7).7
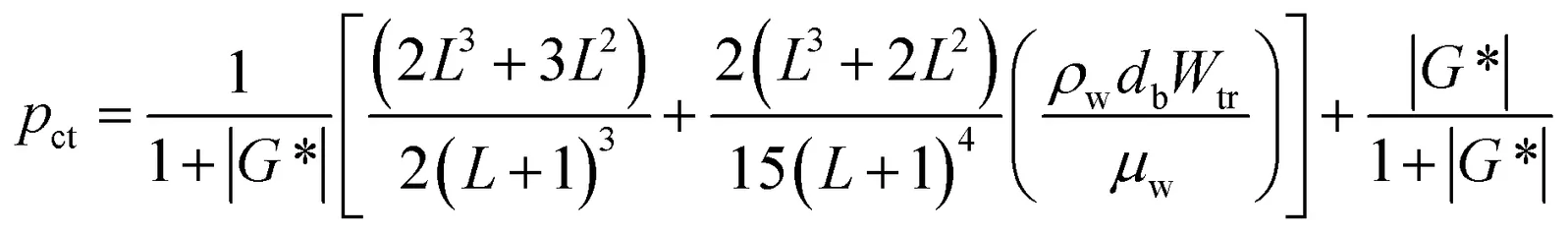


Therefore, by combining eqn (3), (11) and (12), the collision probability between oil droplets and bubbles in the air-flotation cyclone can be calculated.

##### 3.1.1.2 Adhesion of oil droplets and bubbles

The collision behavior between oil droplets and bubbles can promote the contact between them. In analyzing the adhesion behavior of oil droplets and bubbles, the adhesion probability is an important parameter, which refers to the percentage of oil particles that adhere to bubbles out of the total number of oil particles that collided. Currently, there are few models directly focused on the adhesion process of oil droplets and bubbles. However, there are many studies on the adhesion of oil droplets to each other and the adhesion of particles to bubbles, which can be useful for reference. In this study, based on the Bloom–Heindel adhesion probability model,^27^ the velocity ratio in the model is corrected (G*), thereby obtaining an improved adhesion probability model for describing the adhesion process between oil droplets and bubbles. The expression is as follows:8
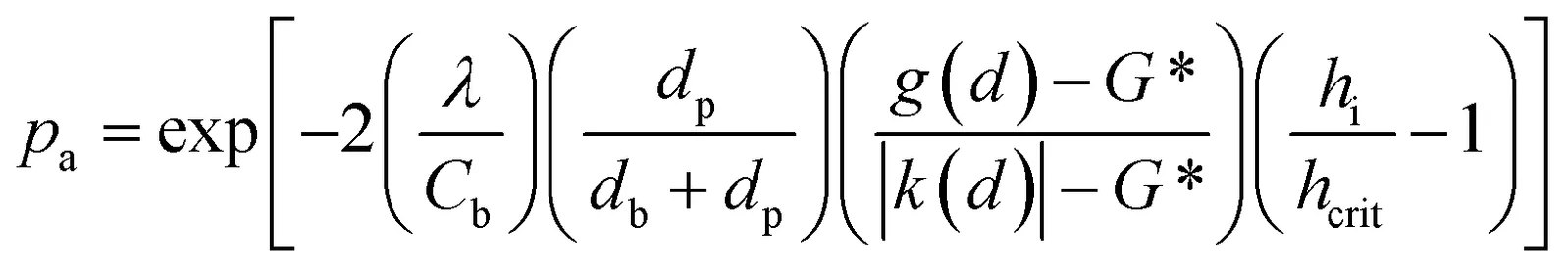
In the equation, *f* is the fluid flow fraction factor. *C*_b_ is the measure of the bubble surface mobility, with a range of 1 to 4, where 1 represents a completely rigid surface and 4 represents an unconstrained bubble surface. *h*_i_ is the initial liquid film thickness, and *h*_crit_ is the critical liquid film thickness. Based on the studies by Moosai, Saththasivam, and others on the adhesion process between bubbles and oil droplets, the critical liquid film thickness is determined to be 0.1 µm. When the liquid film thickness reaches 0.1 µm, it is assumed that the oil droplet has adhered to the bubble surface.

##### 3.1.1.3 Detachment of oil droplets and bubbles

The stable adhesion of an oil droplet to the surface of a bubble depends on the adhesive force between the oil droplet and the bubble, as well as the external stress provided by the environment. When the adhesive force exceeds the sum of the external stresses, the oil droplet can remain stably attached to the bubble surface. Otherwise, the droplet detaches from the bubble surface. Schulze^32^ introduced the Bond number to calculate the stability probability of oil droplets and bubbles, which was later modified by Bloom and Heindel. The expression is as follows:9
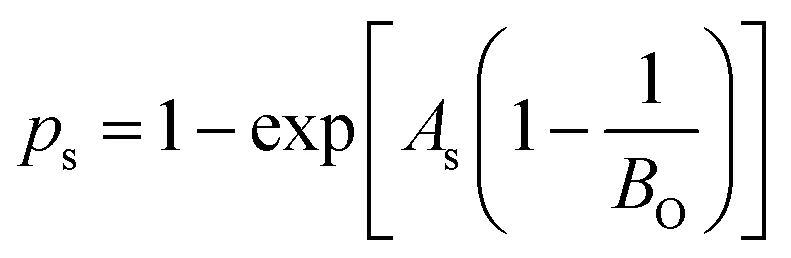
In the equation, *A*_s_ is a constant with a value of 0.5.

Additionally, *B*_o_ is the bond number, which is defined as the ratio of the detachment force to the adhesion force. This is also the basis for accurately calculating the stability probability. When an oil droplet adheres to a bubble, the forces acting on it include: capillary force, hydrostatic pressure, centripetal buoyancy, inertial centrifugal force, mechanical acceleration force, and the force triggered by the bubble interface tension. When the oil droplet adheres to the surface of the bubble, the centripetal buoyancy, inertial centrifugal force, mechanical acceleration force, and the force triggered by the bubble interface tension are the detachment forces, while the capillary force and hydrostatic pressure are the adhesion forces. Based on the definition of the bond number, the Bond number when a mineral particle adheres to the surface of a bubble can be obtained as follows:10
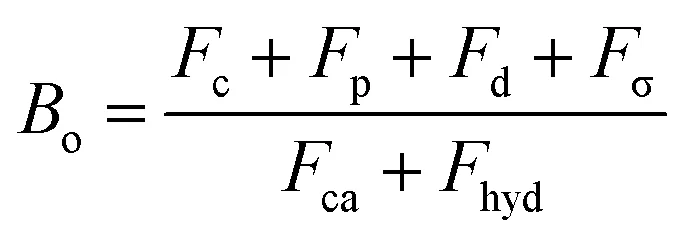
In the equation, *F*_ca_, *F*_hyd_, *F*_c_, *F*_p_, *F*_d_, and *F*_σ_ represent the capillary force, hydrostatic pressure, centripetal buoyancy, inertial centrifugal force, mechanical acceleration force, and the force triggered by the bubble interface tension, respectively. The detailed expressions of the adhesion and detachment forces are provided in Appendix A.

#### 3.1.2 Control equations for device numerical simulation

The CFD model was established by solving the continuity and momentum conservation equations for a viscous incompressible multiphase flow. Bubble-droplet collision, attachment, and stability effects were introduced through user-defined source terms.

#### 3.1.3 Turbulence model

The RNG *k*–*ε* turbulence model was adopted for the numerical simulations in this study. The internal flow field of the air-flotation cyclone is characterized by pronounced streamline curvature, rotational shear, and strong anisotropic turbulence, which cannot be adequately captured by the standard *k*–*ε* model because of its reliance on the isotropic eddy-viscosity assumption. Compared with the standard *k*–*ε* model, the RNG *k*–*ε* model introduces an additional strain-rate correction term derived from renormalization group theory, thereby improving its capability to predict rapidly strained and swirling turbulent flows. Although Reynolds stress models can provide a more detailed representation of turbulence anisotropy, their substantially higher computational cost makes them unsuitable for the repeated simulations required for orthogonal experimental design and neural network dataset construction. Therefore, the RNG *k*–*ε* model provides an appropriate compromise between numerical accuracy and computational efficiency, making it suitable for the multi-parameter optimization framework employed in this study.

#### 3.1.4 Numerical Solution Method

##### 3.1.4.1 Boundary conditions

Based on the flow characteristics inside the device, the inlet boundary condition is set to a velocity inlet condition, and the phase volume fractions are assigned according to actual operating parameters. The outlet boundary conditions are configured based on the split ratio parameters, and a fully developed flow assumption is used, meaning the axial gradient of flow parameters is assumed to approach zero. For solid wall boundaries, a no-slip boundary condition is applied, and the normal component of the velocity gradient is set to zero. The wall function method is used to model the boundary layer, and the turbulent dissipation rate is corrected using the enhanced wall treatment method.

##### 3.1.4.2 Solution method

The transport equations were solved by using the ANSYS Workbench. Bubble–droplet collision, attachment and stability are modeled through user-defined mass source terms. For the discretization of the convection terms, the second-order upwind difference scheme (QUICK scheme) is preferred due to its advantage in capturing flow field curvature. The pressure–velocity coupling solution strategy developed jointly by Patankar and Spalding, based on the pressure correction principle, uses the SIMPLE algorithm. During the calculation process, a relaxation factor is introduced, and the pressure correction and velocity correction terms are iteratively updated to approach the converged solution of the pressure and velocity fields.

#### 3.1.5 Grid for the model

**Fig. 4:**
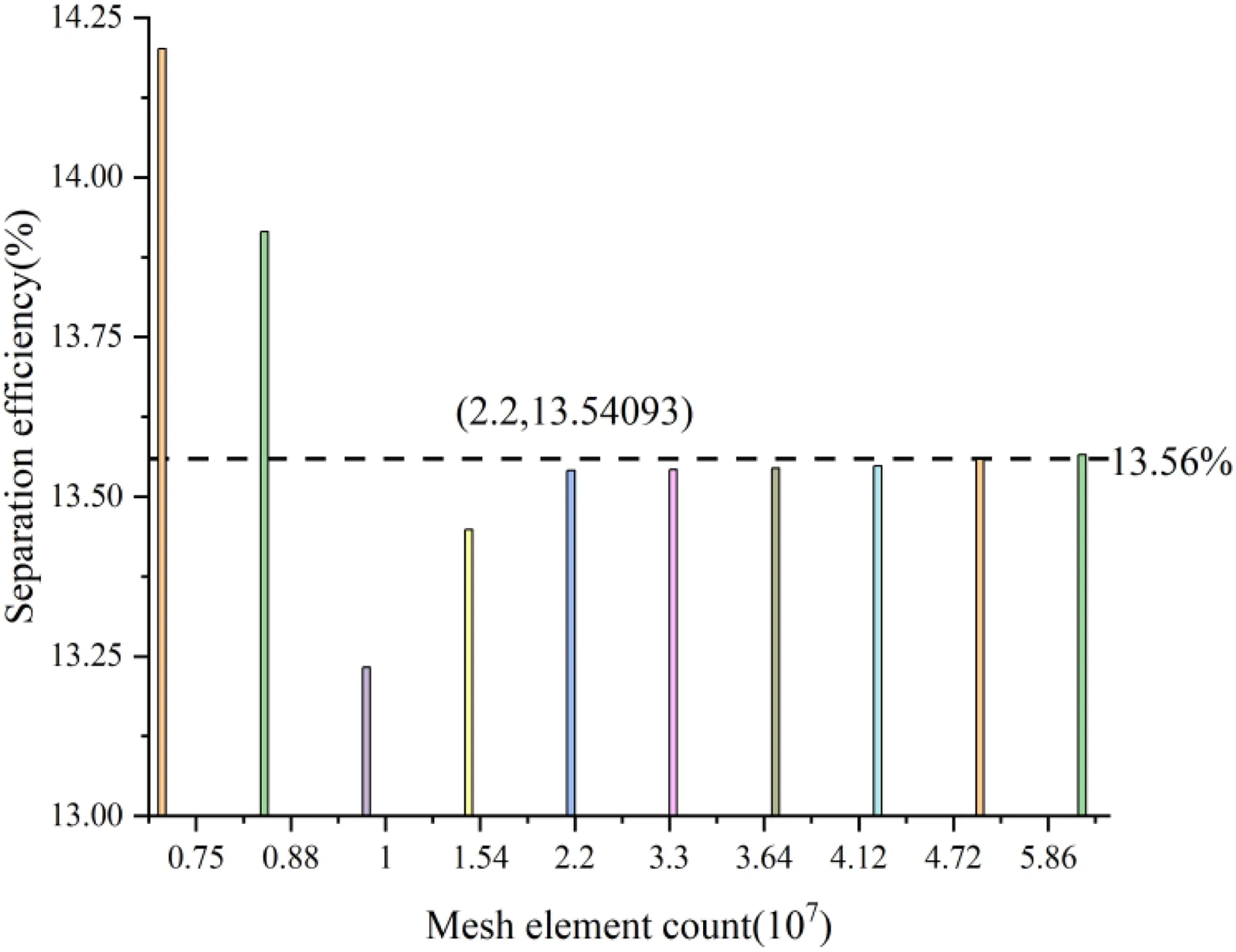
Grid-independent verification.

Considering the axisymmetric geometry of the cyclone separator, an O-type structured mesh was adopted to improve mesh quality and computational efficiency. A grid-independence test was then performed using four mesh systems with approximately 0.75, 1.00, 2.20, and 5.86 million cells. Separation efficiency was selected as the evaluation criterion, and the results are shown in Fig. 4. The predicted separation efficiency was strongly affected by mesh resolution when the cell number was below 2.20 million. This indicates that the local vortex core and near-wall shear structures were not sufficiently resolved on coarser meshes. When the cell number reached 2.20 million or higher, the calculated efficiency became stable and agreed well with the field measurements. Further mesh refinement produced only minor changes in the predicted results but markedly increased the computational cost. Therefore, the mesh with 2.20 million cells was selected for the subsequent simulations. This mesh provides a suitable balance between numerical accuracy and computational efficiency. It also offers a quantitative basis for reducing the uncertainty associated with spatial discretization.

#### 3.1.6 Model validation

**Fig. 5:**
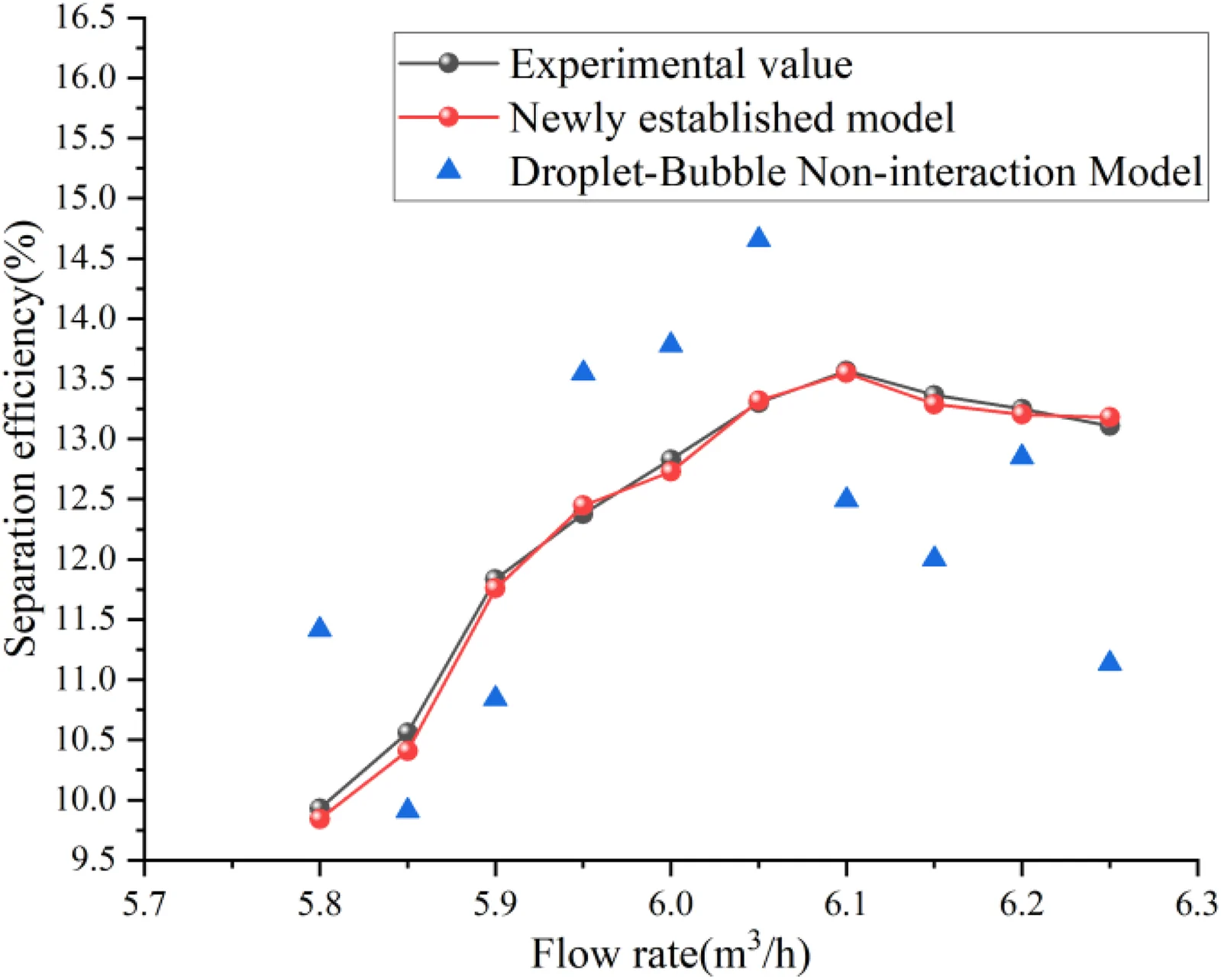
Comparison between simulated and measured separation efficiencies for model validation.

Fig. 5 compares the simulated and measured separation efficiencies under representative field operating conditions. The field tests were conducted at an average inlet oil concentration of 360 mg L^−1^, a median inlet oil droplet size of 50.37 µm, and a treatment flow rate of 5.80–6.25 m^3^ h^−1^. The modified model shows good agreement with the field measurements. The average deviation is 1.2%. In contrast, when bubble-droplet interactions are not considered, the model error increases to approximately 15%. This comparison indicates that the modified interaction model can capture the main physical trend of bubble-assisted oil migration in the cyclone. These results support the applicability of the proposed numerical model for evaluating the performance of air-flotation cyclone enhanced oil-water separation. However, long-term repeated offshore field tests under identical conditions remain difficult to conduct. Therefore, the present validation should be regarded as field-oriented model calibration rather than exhaustive external validation.

## 4 Results and discussion

The specific structural parameters of the current cyclone separator are given in Table 2.4. According to on-site experimental results, when the average droplet size inside the cyclone is 50.37 µm and the average inlet oil concentration is 360 mg L^−1^, the cyclone's treatment capacity is between 5.80 and 6.25 m^3^ h^−1^. The physical properties of the oil and water phases are set based on the measured values at 53 °C.

### 4.1. 1Flow field characteristics inside the cyclone separator

**Fig. 6:**
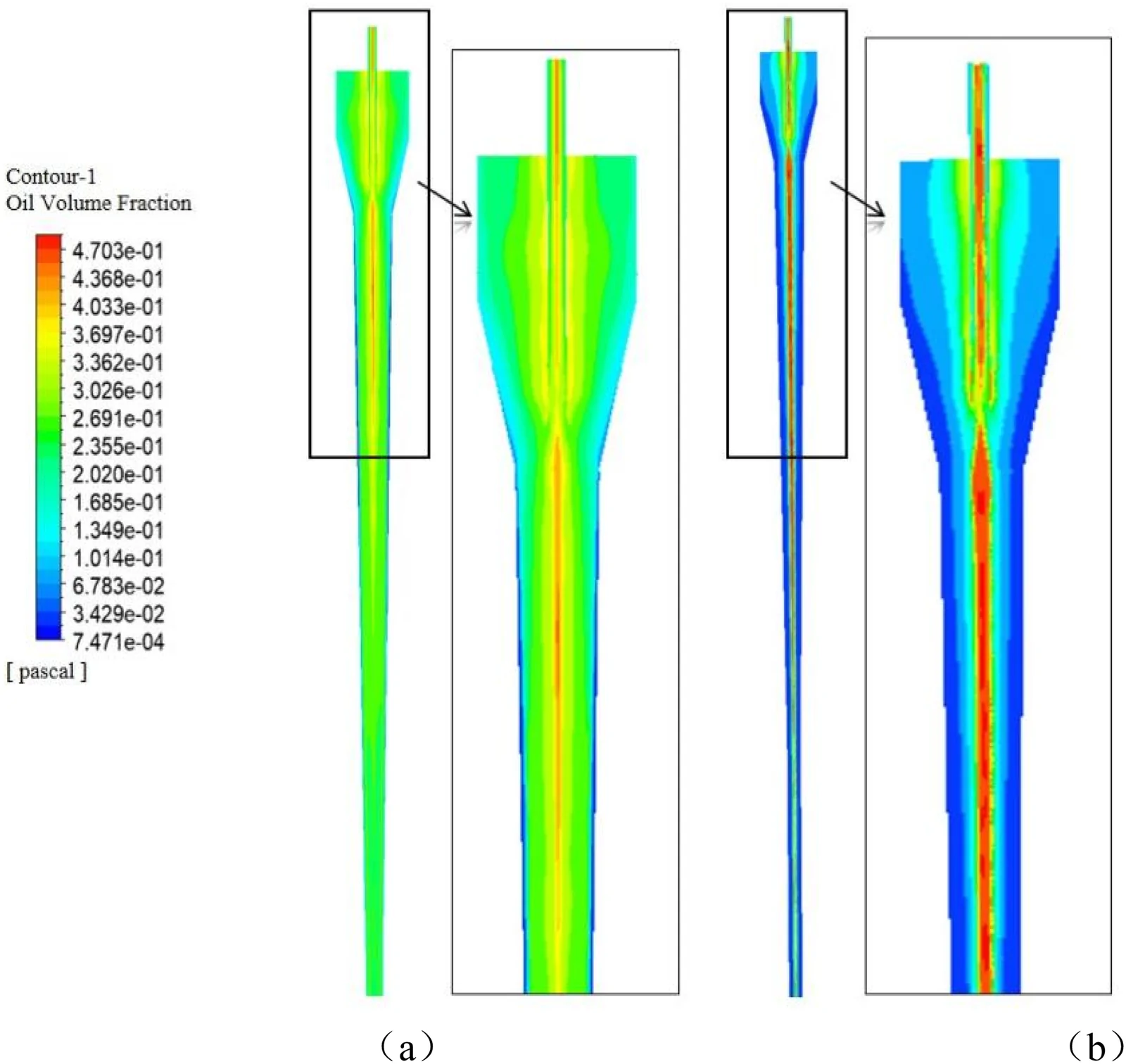
Volume concentration distribution of oil phase in the cyclone tube (a) before gas injection (b) after gas injection.

Fig. 6(a) shows the oil phase concentration distribution in the axial cross-section of the cyclone separator before aeration, while Fig. 6(b) shows the oil phase concentration distribution in the same cross-section after aeration. From the two figures, it can be observed that the lowest oil concentration occurs at the wall, while the highest oil concentration is found in the central regions of the large and small cone sections, with the densest region located in the transition zone between the large and small cones. This not only indicates that the cone section is crucial for oil-water separation but also suggests that the oil-water mixture can achieve effective separation of the two phases under these operating conditions.

Additionally, the comparison between the two figures shows that aeration, which increases the density difference, is beneficial for improving the oil-water separation efficiency. The sharp increase in separation efficiency after aeration is mainly attributed to the formation of bubble-oil droplet composite particles. Because the density of heavy oil is close to that of water, the density difference driving conventional centrifugal separation is very small, resulting in a low baseline separation efficiency. Once micro-bubbles attach to oil droplets, the apparent density of the composite particles decreases significantly. This enlarged effective density difference strengthens the centripetal buoyancy and promotes the migration of the oil phase toward the central overflow region. Therefore, gas injection does not simply disturb the flow field; rather, it reconstructs the separation mechanism by changing the apparent physical properties of the dispersed oil phase.

### 4.2 Effect of overflow outlet position

**Fig. 7:**
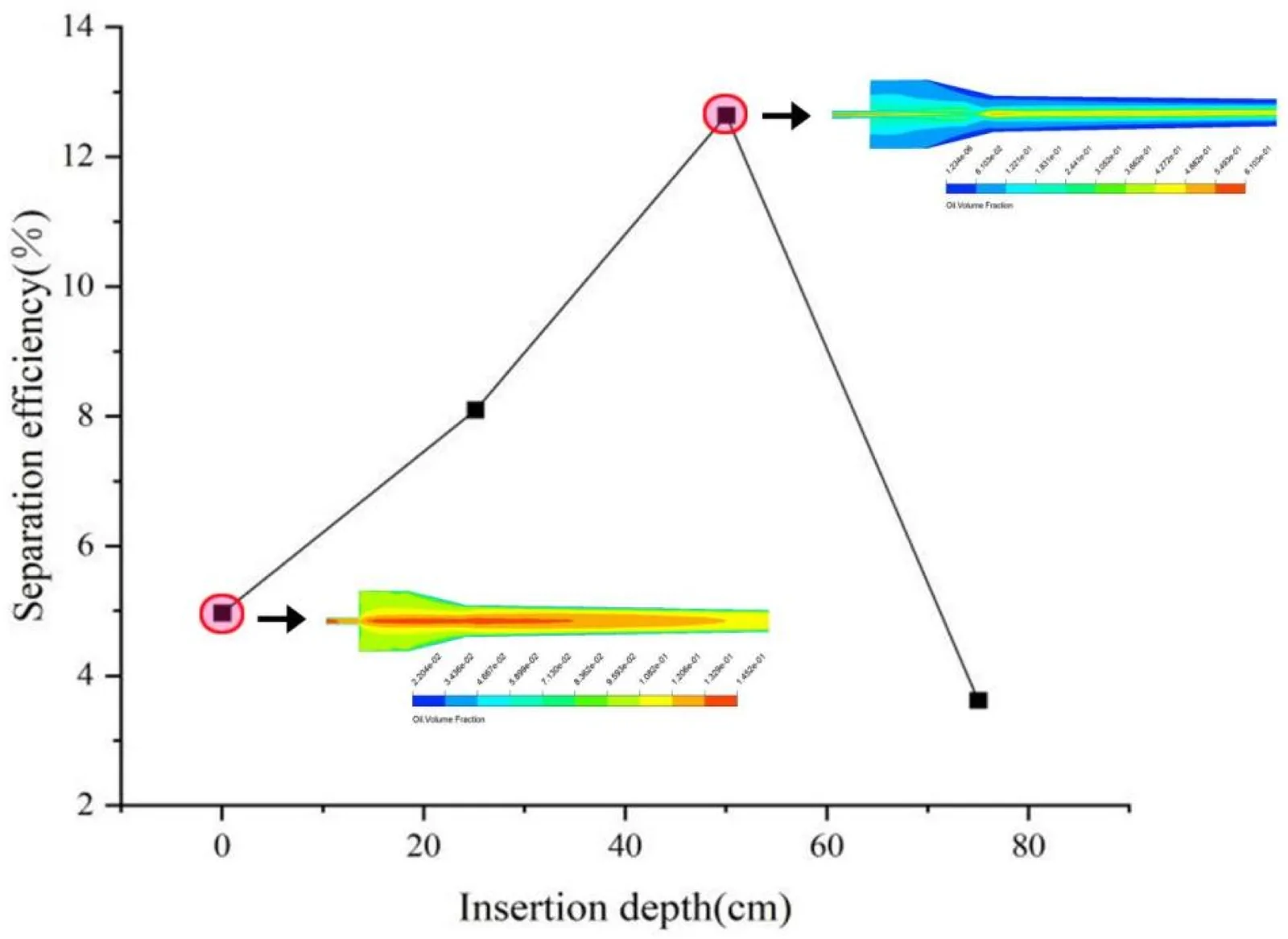
Effect of the depth distance of the overflow port on the separation efficiency of the hydrocyclone.

Simulations were conducted by varying the depth of the overflow outlet's penetration into the cyclone separator, starting from 0. In analyzing the effect of the overflow outlet position on separation efficiency, other structural and operational parameters remained consistent with the field conditions of the oil field. Fig. 7 shows the effect of different depths of overflow outlet penetration on separation efficiency. At a depth of 25 mm, the overflow outlet is located in the cylindrical section; at 50 mm, it is in the large cone section; and at 75 mm, it is in the small cone section. From the figure, it can be seen that when the overflow outlet is positioned deeper into the cylindrical and large cone sections, the oil-water separation efficiency increases.

However, when the overflow outlet is located deeper into the small cone section, the oil-water separation efficiency decreases. The flow field structure in the small cone section is complex, with strong velocity gradients and pressure changes. The deeper penetration of the overflow outlet significantly disturbs the local flow field, disrupting the normal migration and aggregation process of the oil droplets. Moreover, this disturbance may trigger vortexes and other unstable flow patterns, causing the force field acting on the droplets to become distorted. As a result, the oil droplets are unable to maintain a stable aggregation state and may even re-disperse, leading to a reduction in the oil-water separation efficiency. The comparison of oil phase concentration distribution is shown in Fig. 7. From the figure, it is evident that after deeper penetration, the oil phase distribution becomes more concentrated, and the aggregation of the oil phase at the overflow outlet significantly increases.

### 4.3 Effect of overflow outlet diameter

**Fig. 8:**
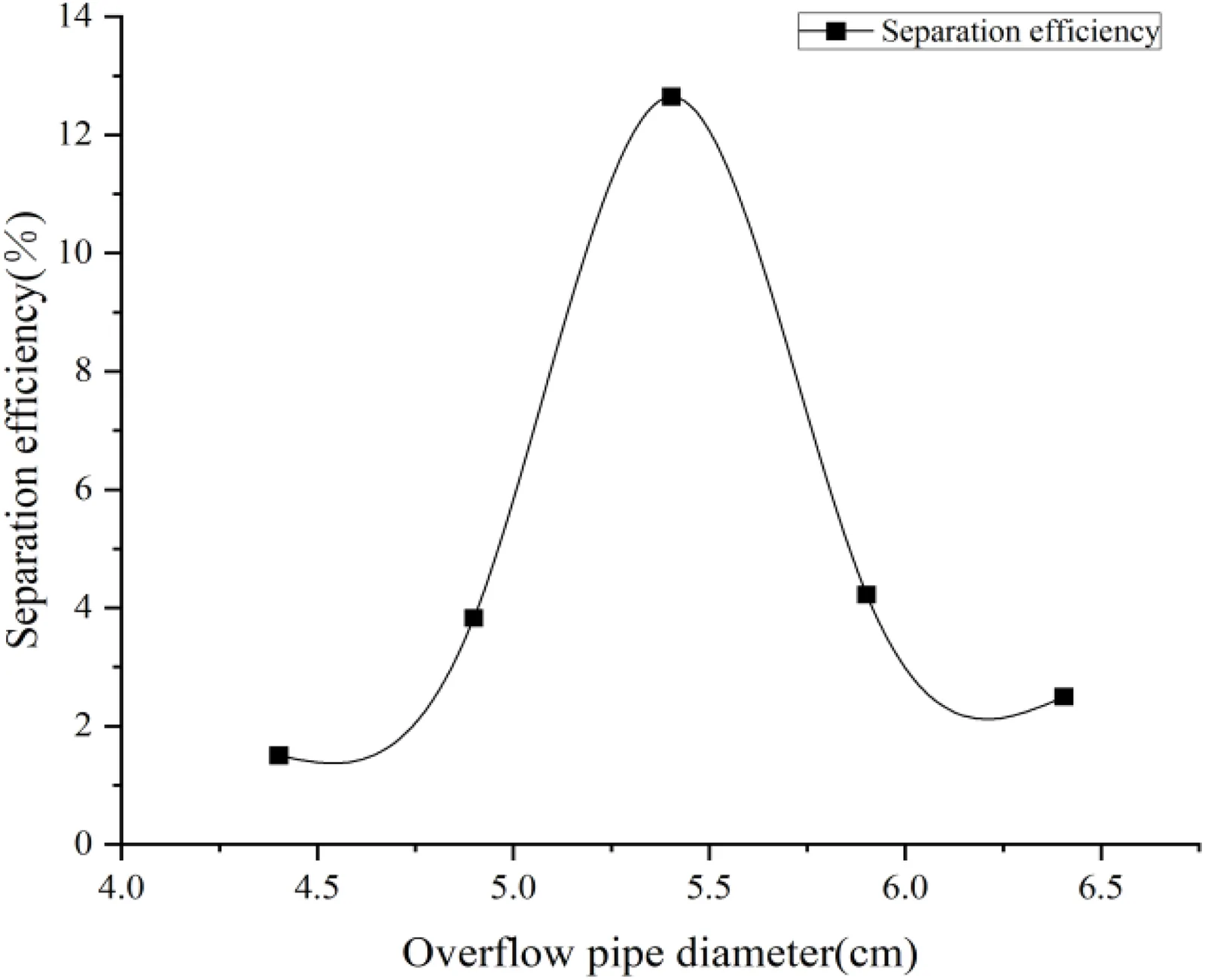
Effect of overflow diameter on the separation efficiency of hydrocyclone.

Fig. 8 shows the variation of the separation efficiency of the hydraulic cyclone with changes in the overflow outlet diameter. When the diameter of the overflow outlet increases from 4.4 mm to 5.4 mm, the separation efficiency gradually rises from 1.52% to 12.66%.

**Fig. 9:**
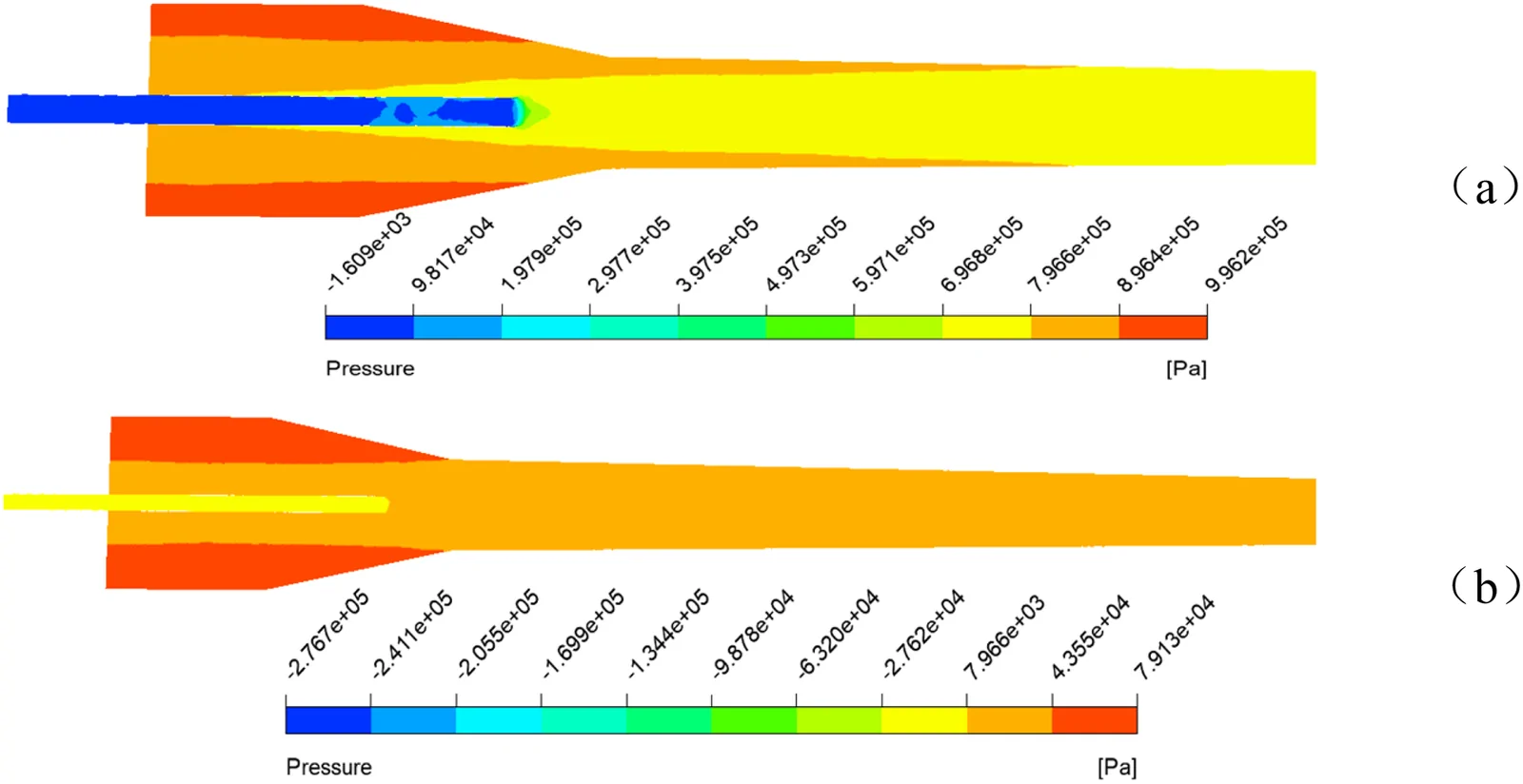
Pressure distribution contour (a) diameter 4.4 mm (b) diameter 6.4 mm.

When the overflow outlet diameter is too small, it results in a significant increase in the loss of hydraulic head.as shown in the pressure distribution map in Fig. 9. When the overflow outlet diameter is 4.4 mm, the pressure at the overflow outlet is significantly lower than that at the overflow outlet with a diameter of 6.4 mm. Additionally, a small overflow outlet diameter makes it difficult for the internal vortex's oil core to flow smoothly out of the overflow outlet. When the overflow outlet diameter increases from 5.4 mm to 6.4 mm, the separation efficiency decreases from 12.66% to 2.52%.

### 4.4 Effect of cylindrical section length

**Fig. 10:**
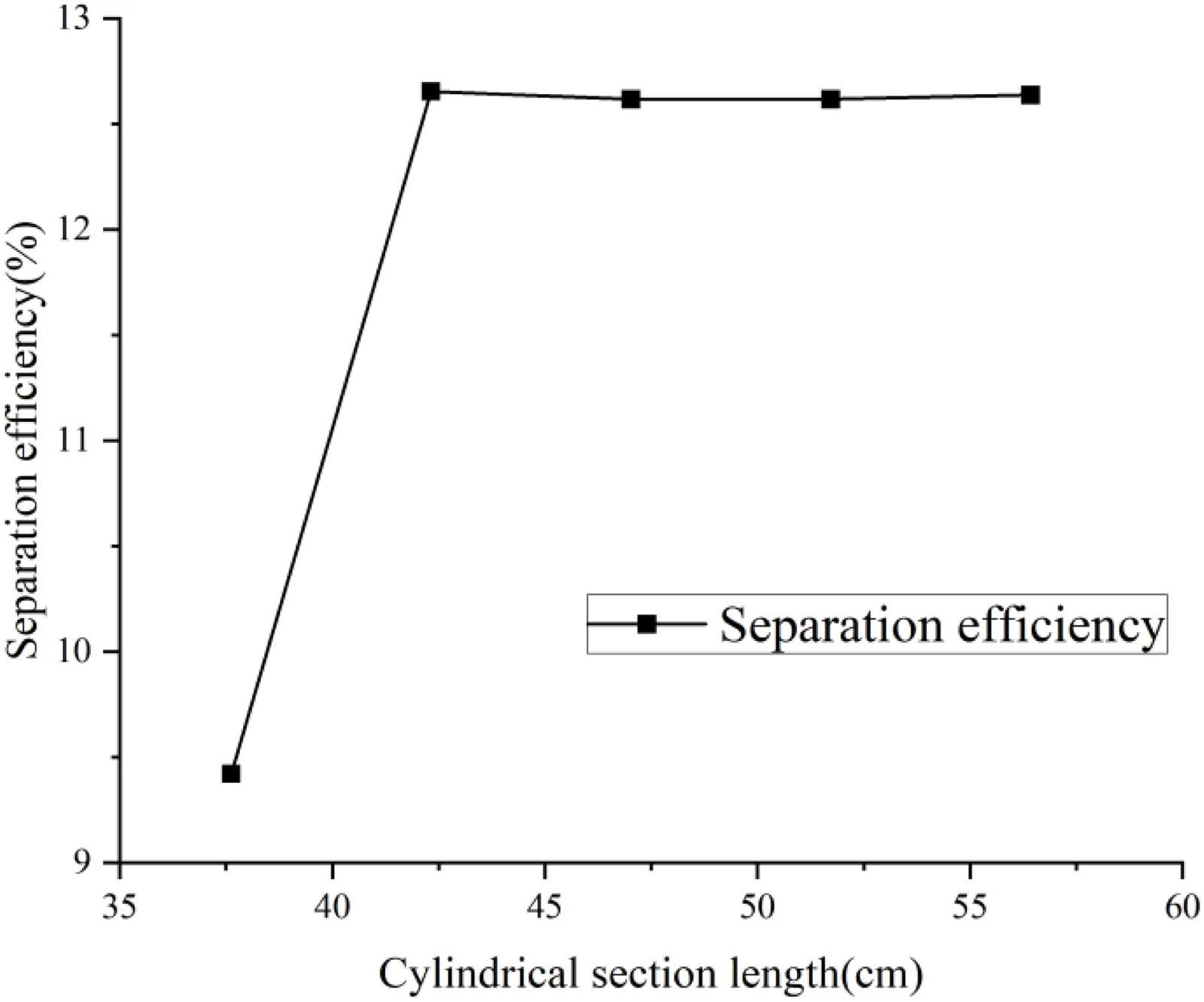
Effect of column length on the separation efficiency of hydrocyclone.

The cylindrical section length (*L*_s_) of the cyclone separator used in the H oil field is 42.5 mm, with a ratio of the cylindrical section diameter (*D*) to the diameter of 0.9. To analyze the impact of different cylindrical section lengths on the separation efficiency and bottom-flow pressure drop, the lengths of the cylindrical section were set to 0.8D, 0.9D, 1.0D, 1.1D, and 1.2D. The effects of different cylindrical section lengths on the separation efficiency and bottom-flow pressure drop of the cyclone separator are shown in Fig. 10.

The results of the numerical simulation reveal the regulation of the cylindrical section axial size on separation efficiency, as shown in Fig. 10. When the cylindrical section length increases to 42.5 mm, the trend of increasing separation efficiency stabilizes, indicating that the axial size of the cylindrical section has a non-dominant influence on separation efficiency.

### 4.5 Effect of cone angle

The separation performance of the hydraulic cyclone is closely related to the cone angle design: a smaller cone angle enhances the centrifugal effect, significantly improving oil-water separation, while a larger cone angle weakens the centrifugal force and increases the likelihood of two-phase mixing.

**Fig. 11:**
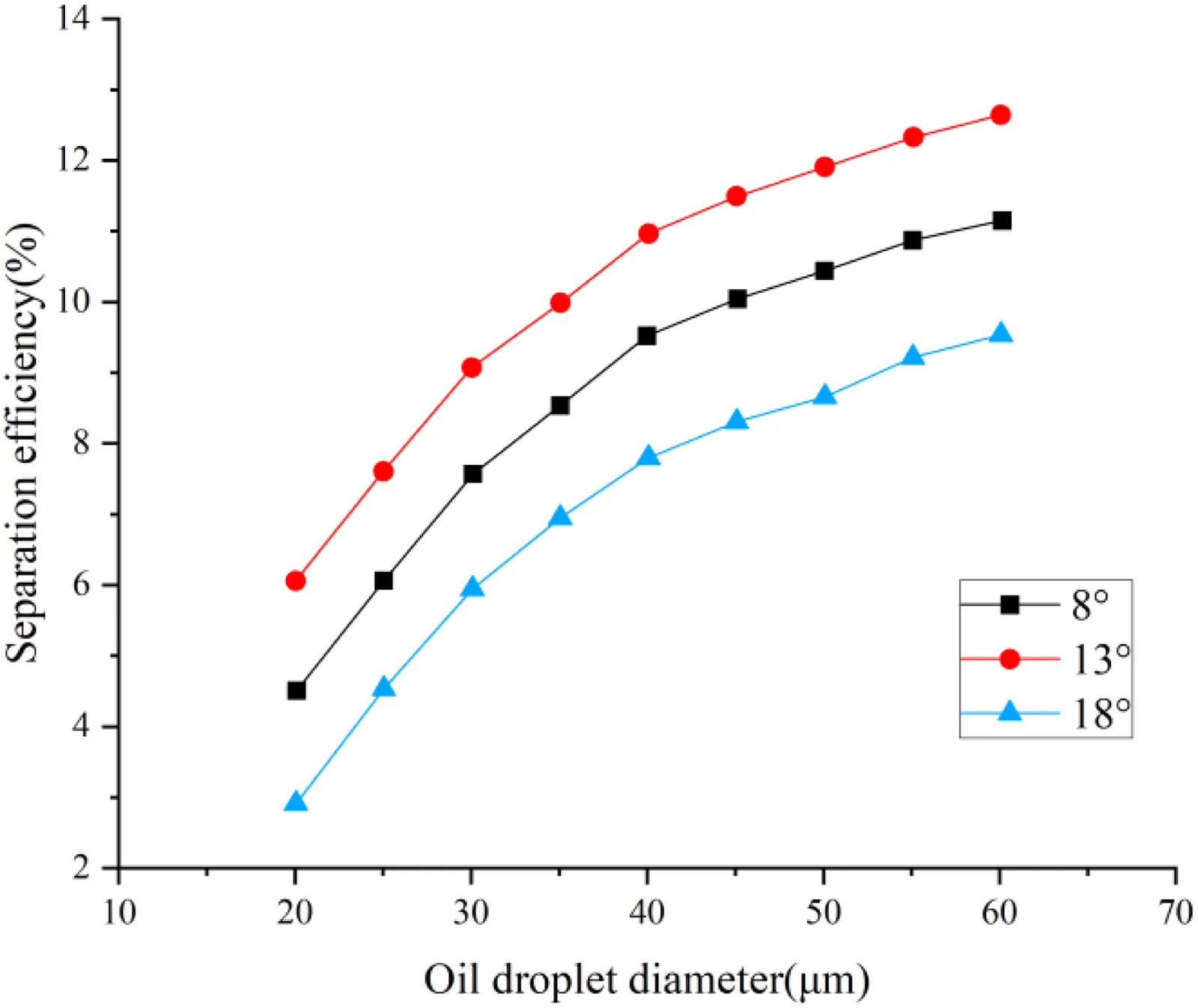
Effect of large vertebral angle angle on the separation efficiency of hydrocyclone.

Fig. 11 shows the particle size – separation efficiency curves for large cone angles of 8°, 13°, and 18°. From Fig. 11, it can be seen that both geometric conditions lead to a decrease of about 3% in the retention efficiency for droplets in the 20–60 µm size range. Among the analyzed large cone angles, a cone angle of 13° provides the best separation efficiency.

**Fig. 12:**
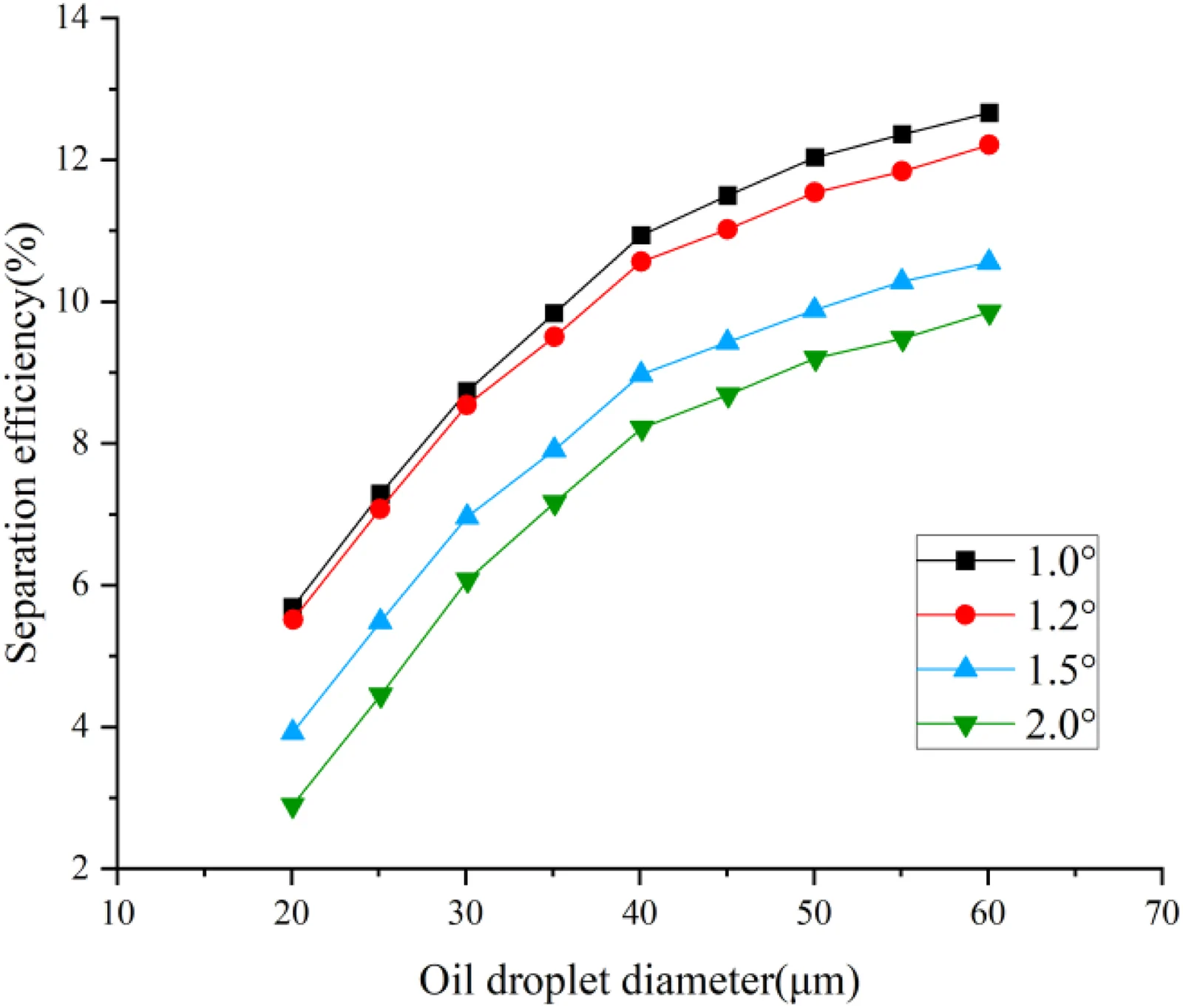
Effect of small vertebral angle on separation efficiency of hydrocyclone.

Fig. 12 shows the particle size – separation efficiency curves for small cone angles of 0.8°, 0.9°, 1.0°, and 1.1°. As shown in Fig. 12, when the droplet size is small, the oil-water separation is most effective with a small cone angle of 1°.

### 4.6 Effect of tailpipe diameter and length

**Fig. 13:**
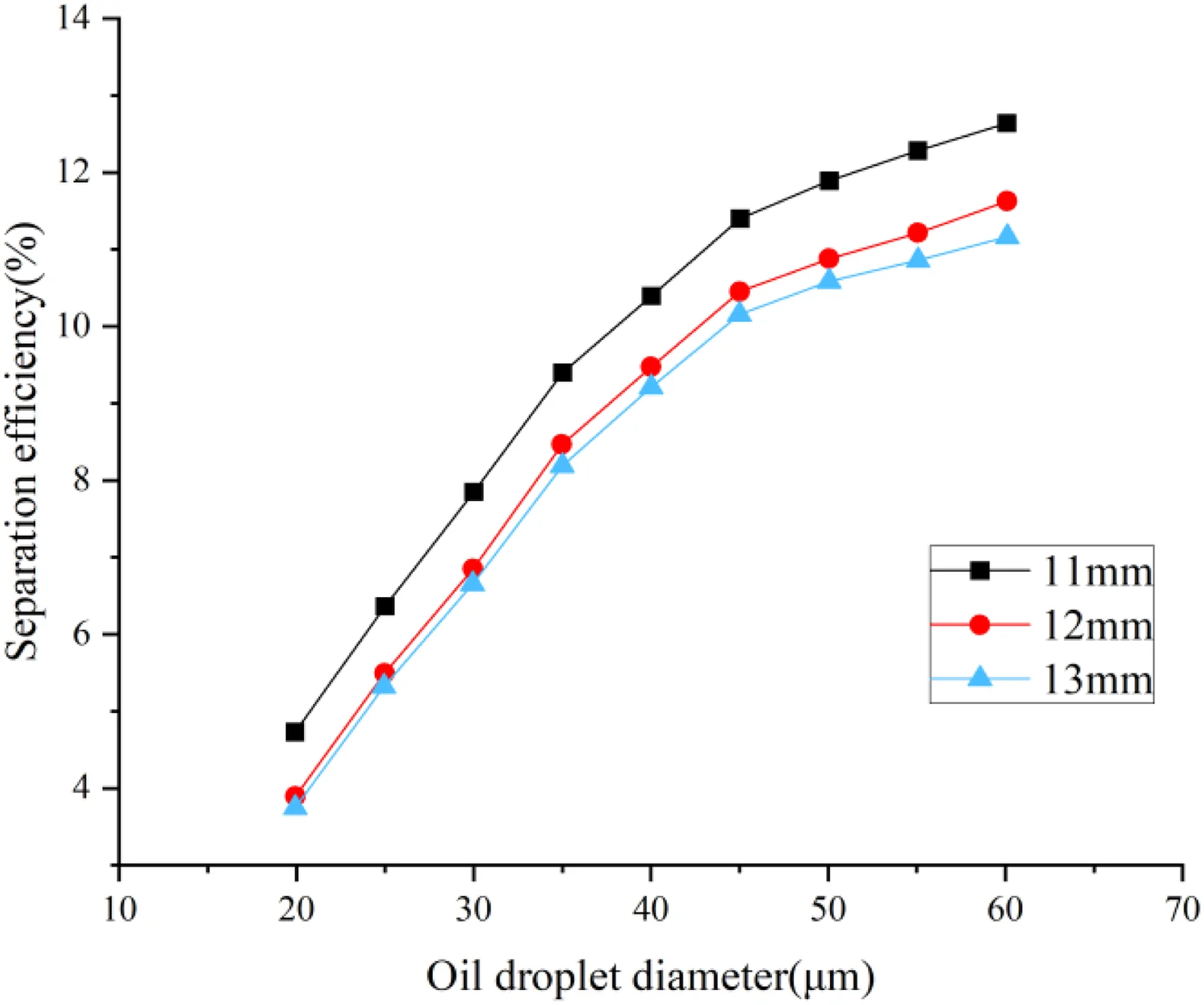
Effect of liner diameter on separation efficiency of hydrocyclone.

The tailpipe diameter of the cyclone separator used in the H oil field is 11 mm, with a length of 442 mm. Fig. 13 shows the particle size efficiency curves corresponding to tailpipe diameters of 11 mm, 12 mm, and 13 mm. When the tailpipe diameter increases from 11 mm to 13 mm, the separation efficiency shows a decreasing trend. However, when the diameter is reduced from 13 mm back to 11 mm, the flow velocity at the bottom outlet increases, and this increase in flow velocity causes a significant change in the static pressure gradient. This pressure differential regulation mechanism enhances the oil phase enrichment efficiency, thereby improving separation performance.

**Fig. 14:**
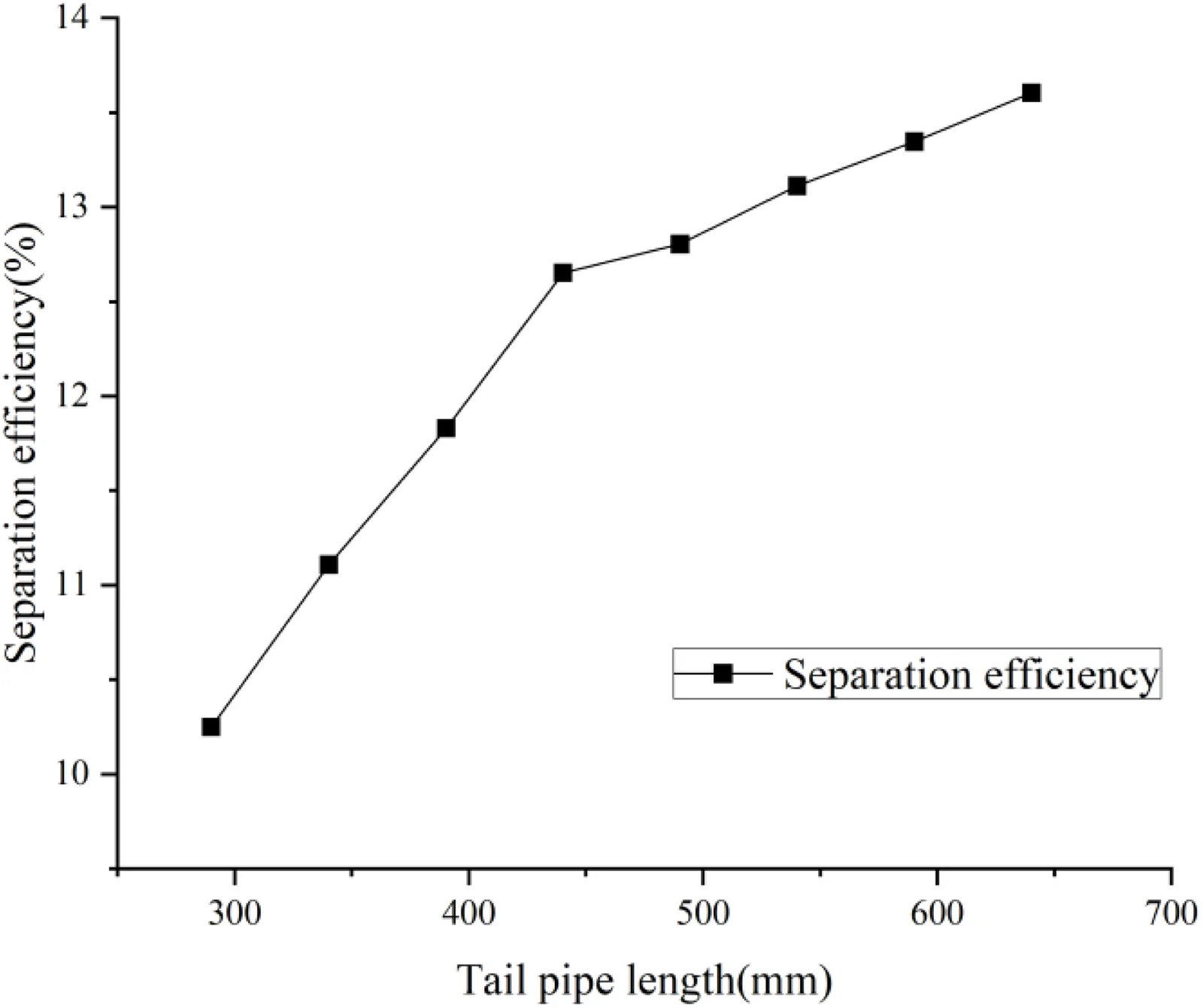
Effect of liner length on the separation efficiency of hydrocyclone.

The regulation of tailpipe length on cyclone separator performance is shown in Fig. 14.When the axial length of the tailpipe increases from the reference value of 290 mm to 640 mm, the separation efficiency initially increases and then stabilizes. Engineering practice shows that the tailpipe parameters used in the H oil field (*L* = 442 mm) achieve an optimized balance between flow field development time and energy efficiency. The design of the tailpipe section used in the cyclone separator is reasonable, ensuring a high separation efficiency.

### 4.7 Effect of flow rate

**Fig. 15:**
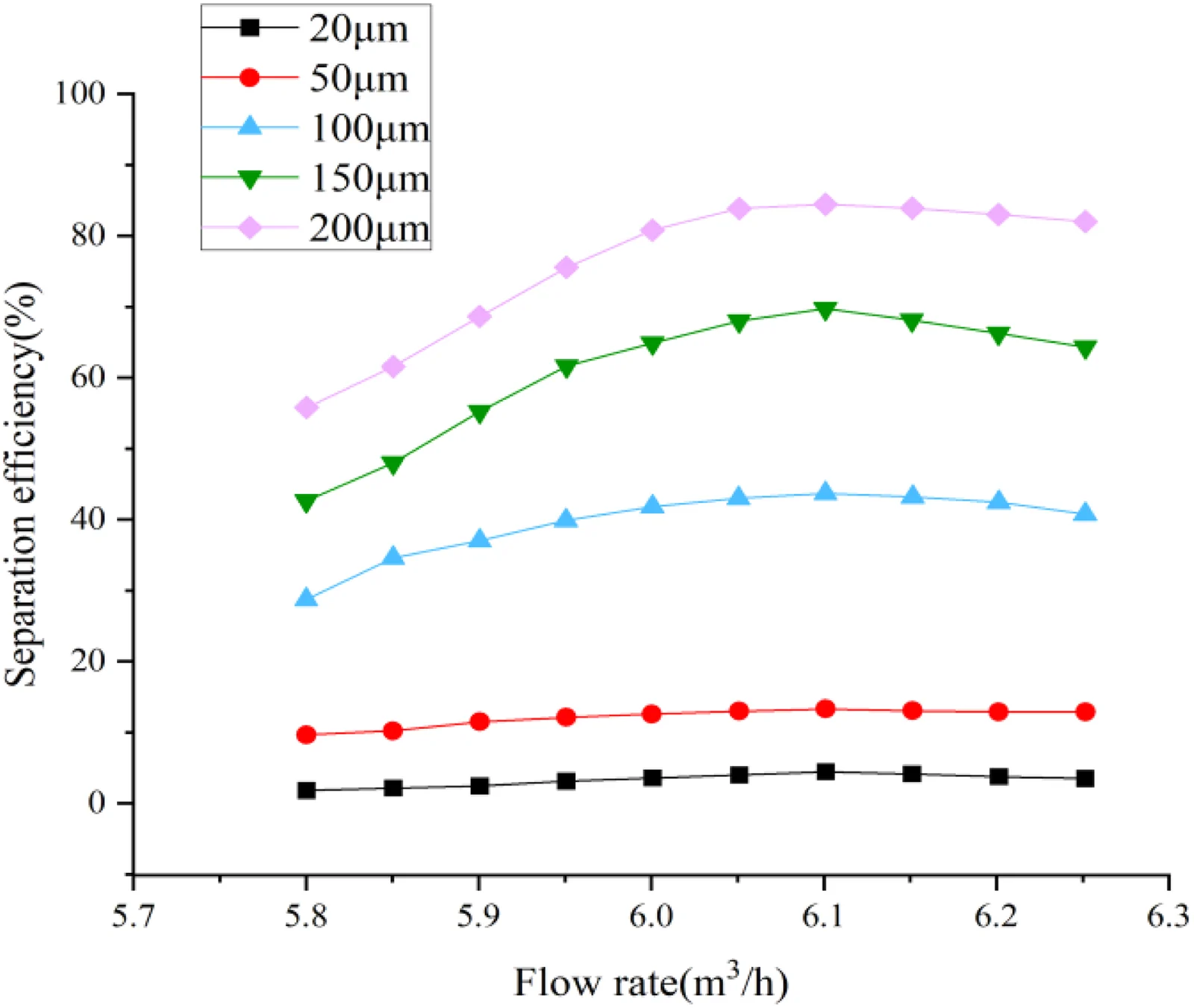
Effect of flow rate on separation efficiency of hydrocyclone.

As shown in Fig. 15, for oil droplets of different particle sizes, the inlet flow rate has a generally increasing and then decreasing effect on the oil-water separation efficiency of the hydrocyclone. For oil droplets with particle sizes of 20 µm and 50 µm, the separation efficiency remains at a relatively low level, with minimal fluctuation as the inlet flow rate changes.

However, for oil droplets with particle sizes of 100 µm, 150 µm, and 200 µm, the separation efficiency increases as the inlet flow rate rises from 5.7 m^3^ h^−1^. When the flow rate reaches a certain level (6.1 m^3^ h^−1^), the separation efficiency for 200 µm oil droplets stabilizes, then slightly decreases; for 150 µm oil droplets, the separation efficiency gradually decreases after reaching a peak; for 100 µm oil droplets, the efficiency increases to a certain level before stabilizing and then gradually declines.

In general, within a certain range, increasing the inlet flow rate helps improve the separation efficiency for larger oil droplet particles. However, once the flow rate exceeds a certain threshold, the separation efficiency no longer increases or may even decrease due to changes in the flow field and other factors.

### 4.8 Effect of particle size

The simulation was conducted for five different bubble sizes: 20 µm, 50 µm, 100 µm, 150 µm, and 200 µm microns. After gas injection, the final results of the cross-simulation are shown in Fig. 12. From Fig. 12, it can be observed that under the basic operating condition without gas injection into the hydrocyclone, as the oil droplet diameter gradually increases from a smaller value, the oil-water separation efficiency shows an upward trend. The separation efficiency increases from below 5% to nearly 85%, with the increase being relatively steady. When gas bubbles of different sizes are injected into the hydrocyclone, the variation of oil-water separation efficiency presents significant differences.

**Fig. 16:**
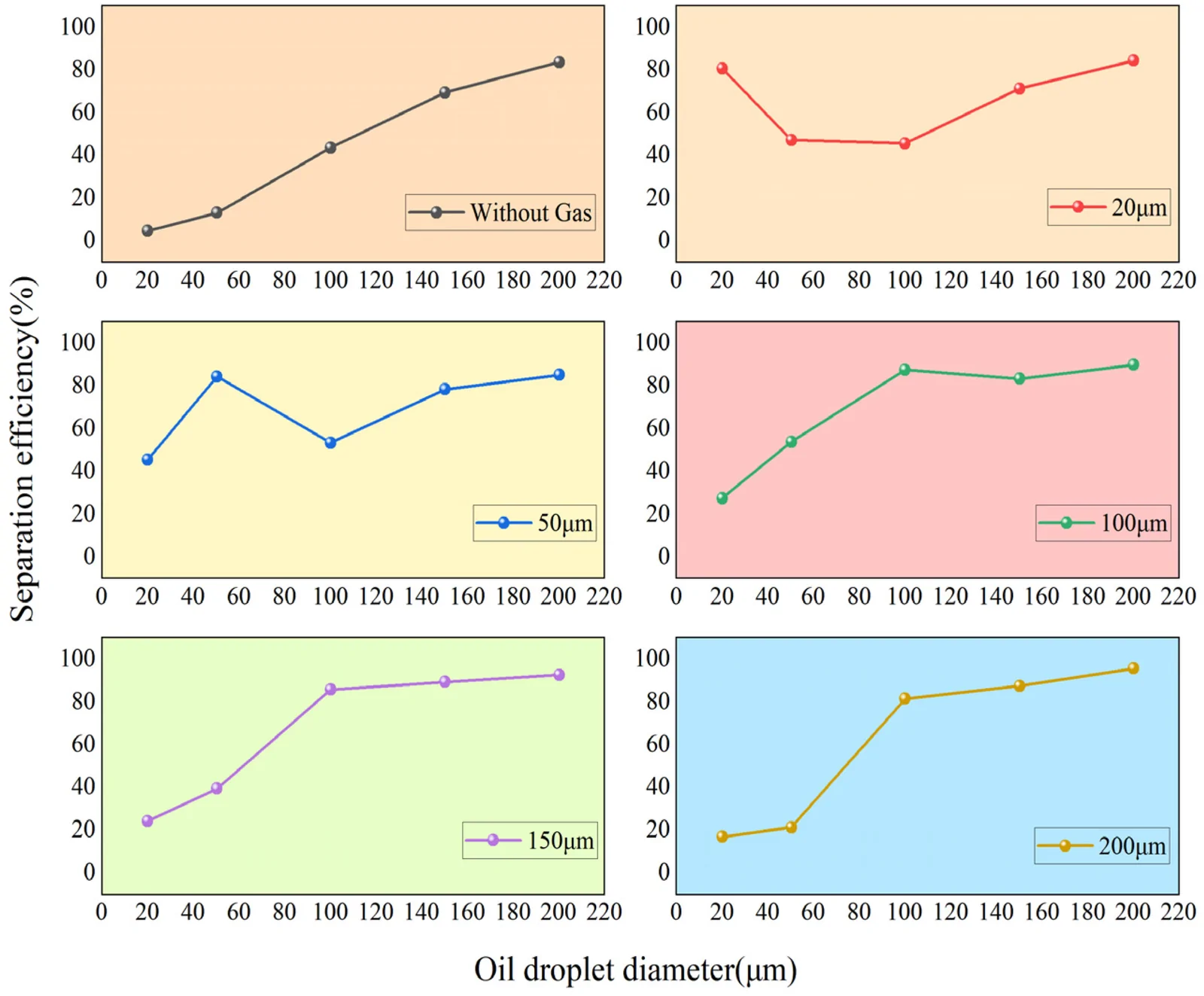
Bubble particle size for hydrocyclone separation efficiency.

Under the condition of gas injection into the hydrocyclone, the separation efficiency improves most noticeably when the oil droplet size matches the injected bubble size. The larger the difference between the two, the weaker the improvement in separation efficiency (Fig. 16).

### 4.9 Impact of oil concentration

**Fig. 17:**
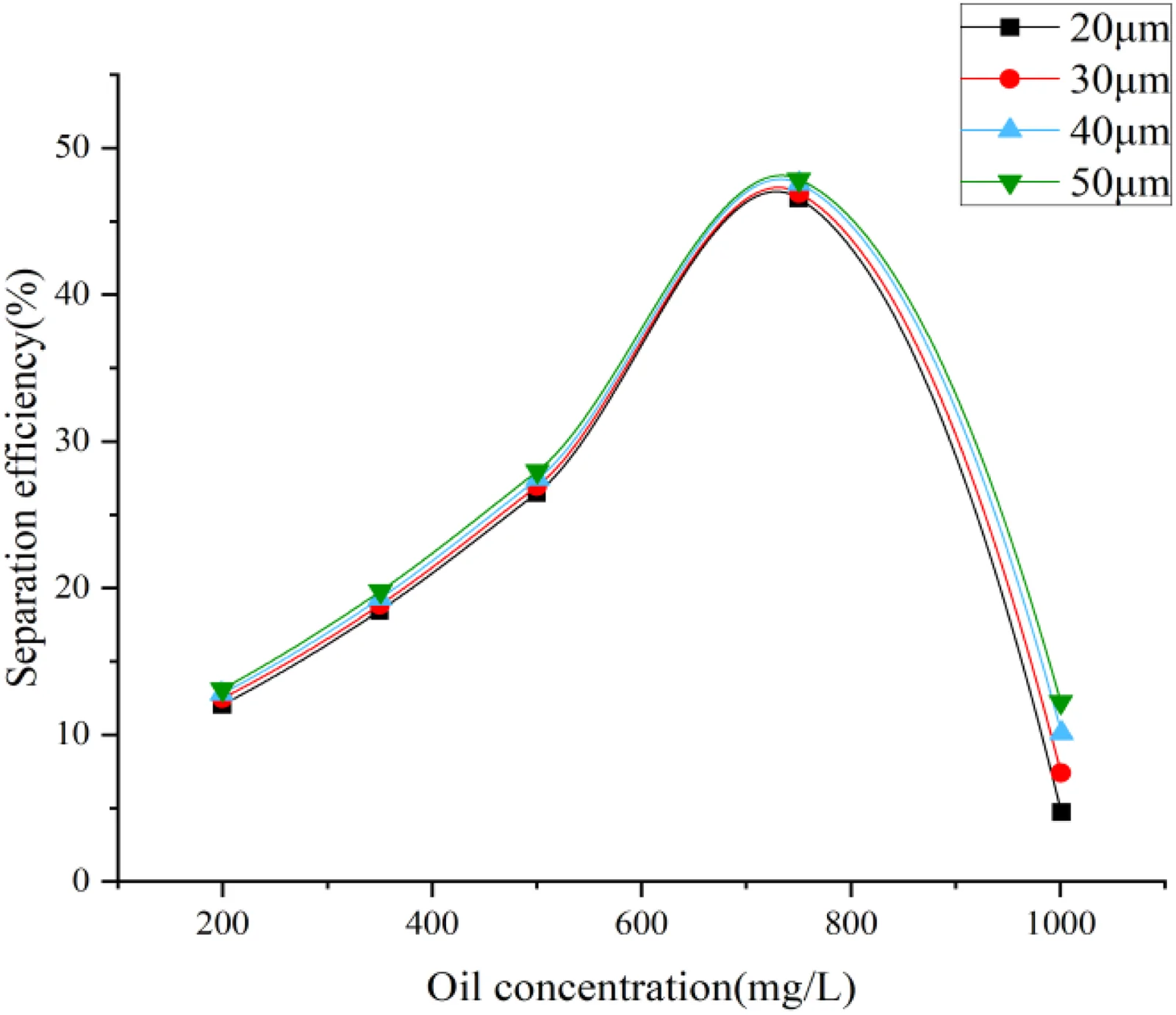
Inlet oil content for hydrocyclone separation efficiency diagram.

By comparing different oil concentrations under equivalent particle size distribution conditions, when the oil concentration increases from 200 mg L^−1^ to 1000 mg L^−1^, the oil-water separation efficiency first increases and then decreases, as shown in Fig. 17. When the oil concentration is between 200 mg L^−1^ and 750 mg L^−1^, the oil-water separation efficiency gradually increases. However, when the inlet oil concentration reaches 1000 mg L^−1^, the oil-water separation efficiency decreases significantly.

## 5 Parameter optimization analysis based on BP neural network

### 5.1 Simulation data collection

Three sets of different data were uniformly selected for orthogonal experiments to collect the data foundation. The experimental factors and their corresponding levels are shown in Table 2.

**Table 2:** Factors and levels of experiments

Factor	Factor name	Units	Level 1	Level 2	Level 3
*A* (Numeric)	Overflow pipe insertion distance	mm	0	25	50
*B* (Numeric)	Overflow pipe diameter	mm	4.4	5.4	6.4
*C* (Numeric)	Cylindrical section length	mm	37.6	42.3	51.7
*D* (Numeric)	Large cone angle	°	8	13	18
*E* (Numeric)	Small cone angle	°	1	1.2	1.5
*F* (Numeric)	Tail pipe diameter	mm	11	12	13
*G* (Numeric)	Inlet flow rate	m^3^ h^−1^	5.8	6.1	6.25
*H* (Numeric)	Bubble particle diameter	µm	20	50	100
*I* (Numeric)	Oil droplet particle size	µm	20	50	100
*J* (Numeric)	Oil concentration	mg L^−1^	350	500	750

Based on the influencing factors identified above, a complete parameter database was established. The single-factor simulation results were first imported and compared. These results were used to determine the key variables and their adjustment ranges. An L27(3^13^) orthogonal array was then generated using SPSS. Ten factors with three levels were included in the design. The factor combinations were added to the simulation database. For each combination, a numerical model was established and solved to obtain the corresponding separation efficiency. The final dataset contained 226 parameter combinations. This number was higher than the minimum data size generally required for neural network construction and training. Therefore, the dataset was considered sufficient for model development and validation. Before model training, all variables were normalized to the interval [0, 1] using the min–max normalization method, expressed as (*x* − *x*_min_)/(*x*_max_ − *x*_min_).

### 5.2 Neural network fitting

#### 5.2.1 Neural network modeling

The separation efficiency of the air-flotation cyclone is governed by the coupling of structural parameters, operating conditions, and multiphase flow behavior. To describe this nonlinear relationship, a BP neural network prediction model was developed using the MATLAB Neural Network Toolbox. The network contained 10 input nodes. These inputs corresponded to overflow pipe insertion distance, overflow pipe diameter, cylindrical section length, large cone angle, small cone angle, tailpipe diameter, inlet flow rate, bubble diameter, oil droplet diameter, and inlet oil concentration. The output node was the oil-water separation efficiency. All input and output data were normalized to the interval [0, 1] before training. A hold-out validation strategy was adopted. The dataset was divided into training, validation, and testing subsets. The validation subset was used to monitor overfitting during training. The testing subset was not involved in model training and was used only for final performance evaluation.

#### 5.2.2 Model training dynamic analysis

The model was trained using the MATLAB neural network toolbox, and Fig. 7 shows the curve of the Mean Squared Error (MSE) for both the training and validation sets as a function of training epochs. The model converged after 27 iterations. During the early stage of training (first 10 epochs), the MSE for the training set dropped sharply from an initial value of 1.7915 to 0.0052004, a decrease of 99.7%, indicating that the network quickly established the basic mapping relationship between input parameters and separation efficiency. It is worth noting that when the iteration reached the 21st epoch, the validation set error showed a local minimum (MSE = 0.0027217), a reduction of three orders of magnitude compared to the initial error, indicating that the network had developed effective generalization ability.

**Fig. 18:**
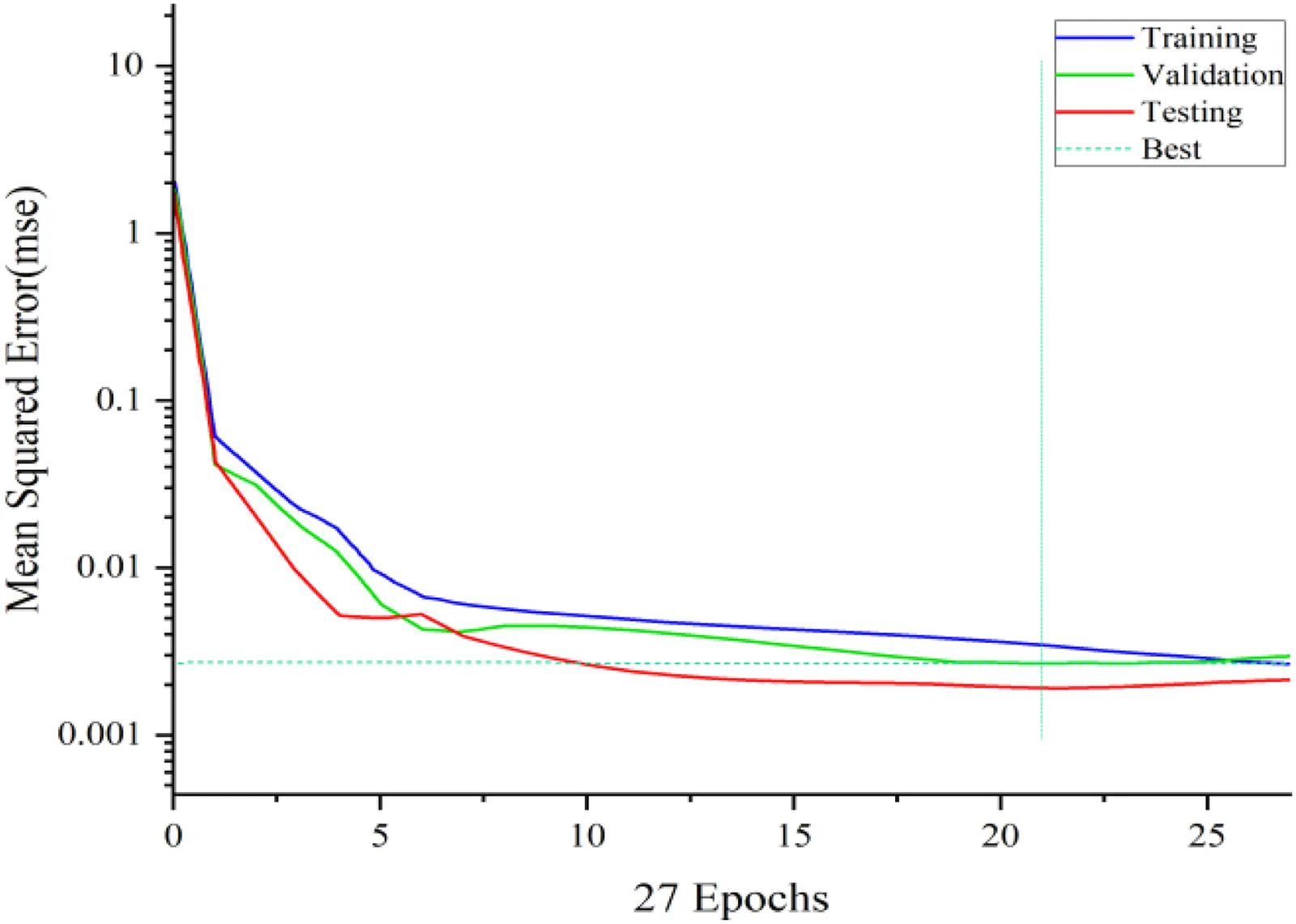
Mean square error performance curve.

As training continued (epochs 10–27), the training set error continued to decrease to 0.00272167, while the validation set error fluctuated within the range of 0.0030 ± 0.0005, showing typical signs of overfitting. To address this, a dynamic early stopping strategy was implemented: when the validation error did not break the historical best value for 6 consecutive iterations, training was stopped immediately. The final model was terminated after 27 epochs, with a training set MSE of 0.00272167 (corresponding to RMSE = 5.2%) and a validation set MSE of 0.00301322 (RMSE = 5.4%), meeting the preset convergence threshold (Fig. 18).

#### 5.2.3 Model performance validation

**Fig. 19:**
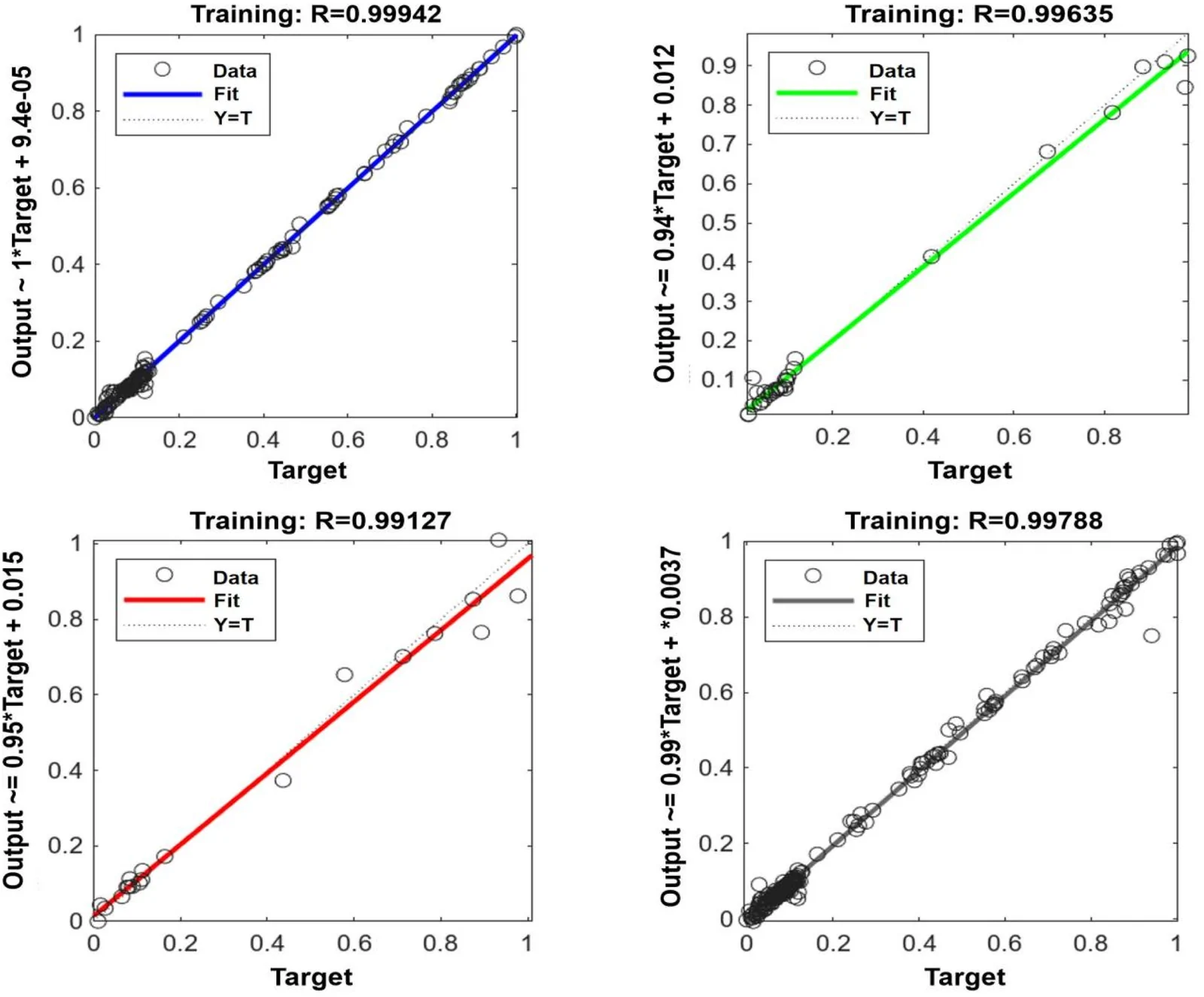
Linear regression fitting.

**Fig. 20:**
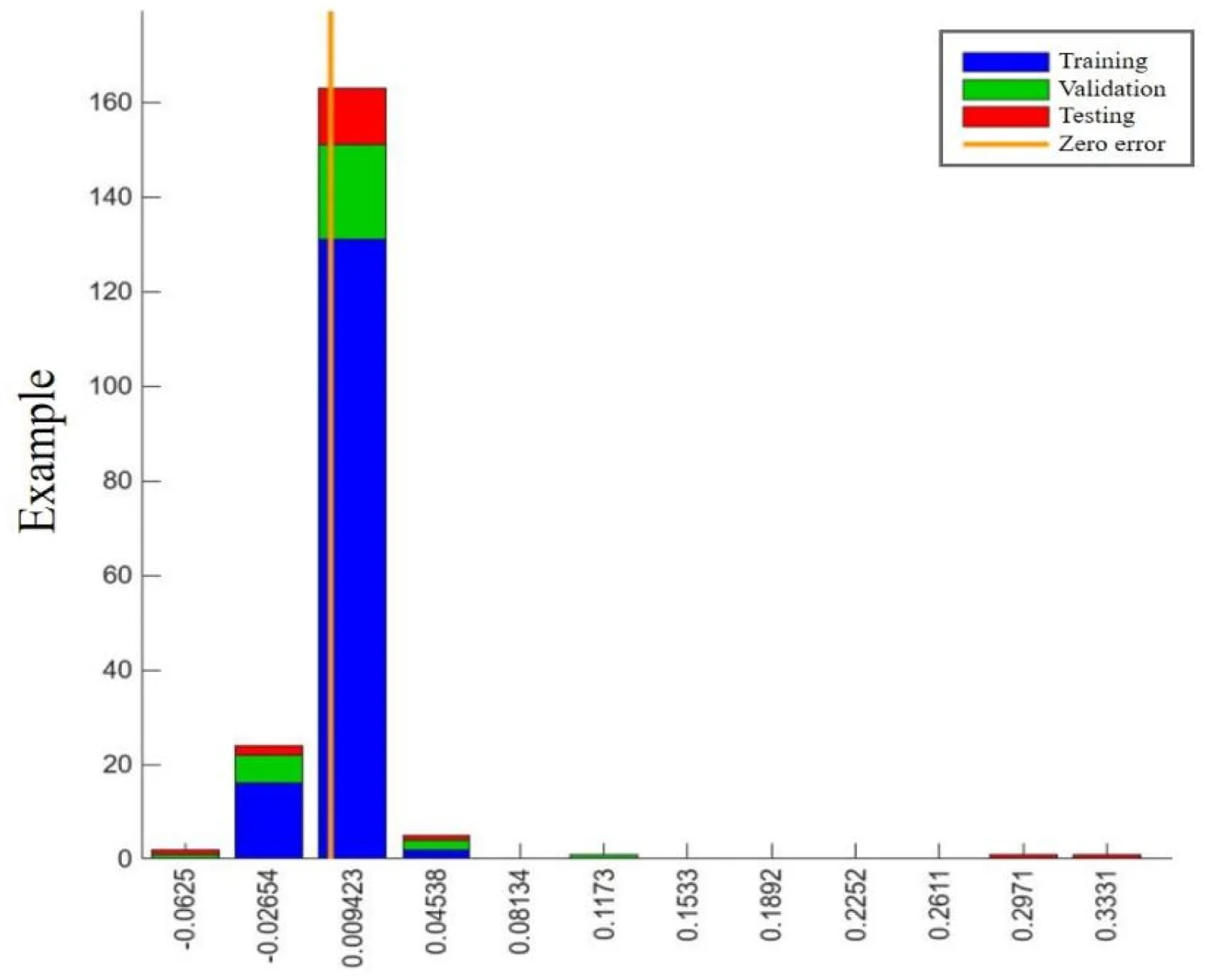
Error histogram.

An independent test set was used to evaluate the generalization ability of the BP neural network. Fig. 19 shows the regression results for the training, validation, testing, and overall datasets. The predicted values are closely distributed along the reference line, (*y* = *x*). The Pearson correlation coefficients of all datasets are higher than 0.99. This indicates that the model can accurately capture the nonlinear relationship between the input parameters and separation efficiency. The quantitative evaluation further confirms the predictive performance of the model. For the test set, the mean squared error is 0.00223785, corresponding to an RMSE of 4.7%. The mean absolute error is 0.009423. As shown in Fig. 20, more than 95% of the prediction errors fall within the ±5% range. Under extreme operating conditions, the absolute prediction error remains below 0.2. These results demonstrate the robustness of the model under complex parameter combinations. The high Pearson correlation coefficient does not imply that overfitting was ignored. During training, a dynamic early-stopping strategy was used. Training was terminated when the validation error failed to decrease for six consecutive epochs. This strategy reduced the risk of memorizing numerical noise in the CFD-generated samples. It should also be noted that the independent test set used in this study was an internal independent dataset derived from the CFD-based orthogonal design. It was not an external dataset from another oilfield. Therefore, further external validation will be required in future pilot-scale applications.

### 5.3 Optimal optimization results and simulation verification

#### 5.3.1 Optimal design parameters

After fitting the data of various factors using the neural network toolbox in MATLAB, the optimal design structure parameters and specific operational parameters for the air-flotation cyclone separator were selected based on the highest separation efficiency results and field data from the H oilfield (where the oil droplet diameter is primarily around 50 µm). Particle swarm optimization was used to obtain the optimal design results, which are shown in Table 3.

**Table 3:** Cyclone tube parameters at the highest separation efficiency

Overflow pipe position	Overflow pipe diameter	Cylindrical section length	Large cone angle	Small cone angle	Tail pipe diameter	Tail pipe length	Inlet flow rate
0 mm	6.4 mm	47 mm	13°	1°	11 mm	440 mm	6.25 m^3^ h^−1^

In the provided optimization results, the optimization plan specifically selected oil droplet diameter and bubble diameter as 50 µm, and the inlet oil concentration as 360 mg L^−1^, to closely match the actual working conditions of the H oilfield. This optimization serves as a guide for subsequent field equipment improvements.

The comparison of separation efficiency before and after optimization of the hydrocyclone structural parameters is shown in Table 4. From the data in the table, it is evident that the separation efficiency significantly improves after gas injection. Furthermore, after optimization using the BP neural network, the separation efficiency increases by more than 10%, indicating a successful optimization.

**Table 4:** Comparison of parameters before and after gas injection and before and after optimization

Scheme comparison	Data source	Separation efficiency
Before gas injection and optimization	Fluent simulation	5.01%
After gas injection but before optimization	Fluent simulation	84.39%
After gas injection and optimization	BP neural network prediction	95.71%

The increase in separation efficiency from 5.01% before gas injection to 84.39% after gas injection should be interpreted as a mechanism-oriented improvement under defined numerical boundary conditions. The low efficiency before gas injection reflects the intrinsic limitation of centrifugal separation for heavy oil droplets. This limitation is mainly caused by the small density difference between heavy oil and water. After gas injection, microbubbles attach to oil droplets and form bubble-droplet aggregates. These aggregates have a much lower apparent density than individual oil droplets. As a result, they can migrate more effectively toward the overflow under centripetal buoyancy. This mechanism explains the large improvement in separation efficiency after gas injection. The optimized efficiency of 95.71% represents a theoretical prediction under field-oriented but idealized simulation conditions. It should not be regarded as a directly validated field value. Further pilot-scale tests are required under fluctuating inlet flow rate, oil concentration, gas injection stability, and droplet-size distribution.

#### 5.3.2 Analysis of optimization results

Based on the data obtained after model development, the specific values for the oil phase mass flow rate at three points were calculated, and the separation efficiency was determined. The final optimized separation efficiency showed a prediction error of less than 0.1%, indicating that the accuracy of the prediction model meets the required standards (Table 5).

**Table 5:** Comparison of parameters before and after injection and before and after optimization

	Overflow oil phase mass flow rate	Inlet oil phase mass flow rate	Underflow oil phase mass flow rate	Separation efficiency
Before gas injection and optimization	0.0156992	0.3136	0.299916	5.006%
After gas injection but before optimization	0.1575892	0.186737	0.054559	84.391%
After gas injection and optimization	0.1507924	0.157554	0.0058864	95.708%

**Fig. 21:**
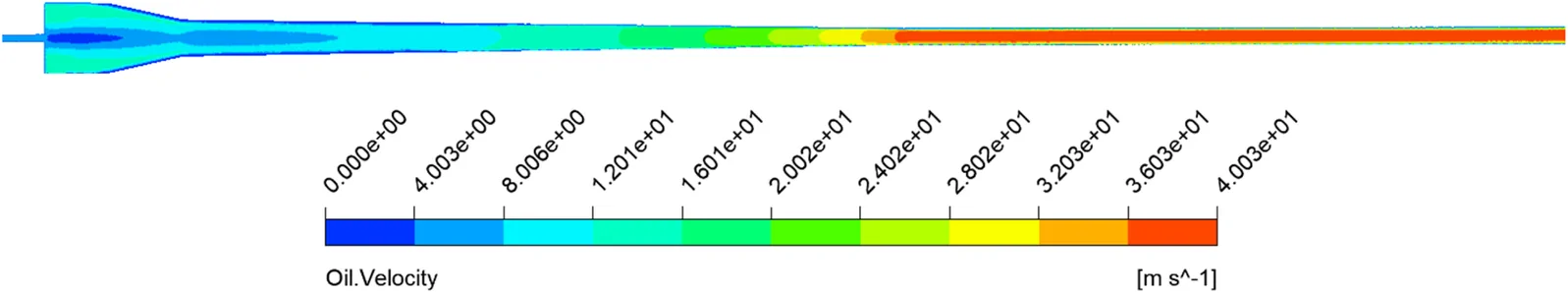
Velocity distribution after optimization.

The velocity distribution after optimization of the cyclone tube is shown in Fig. 21.

**Fig. 22:**
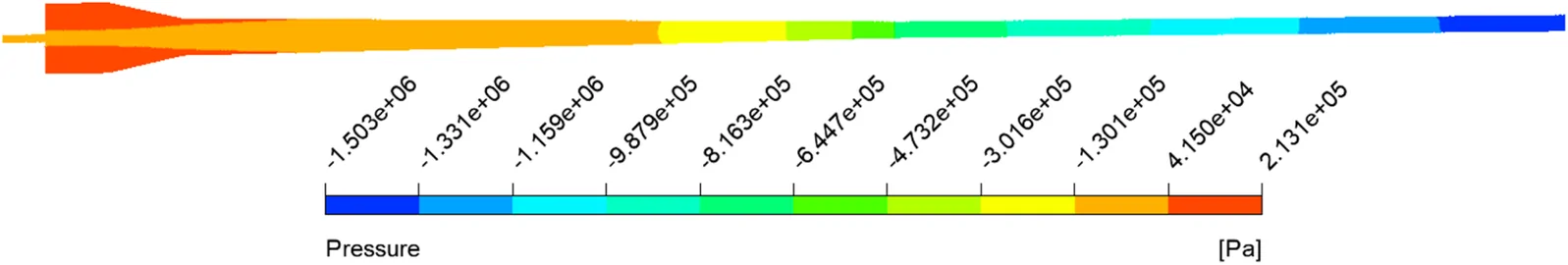
Optimized pressure distribution.

The pressure distribution after optimization of the cyclone tube is shown in Fig. 22.

From Fig. 22, it can be observed that after optimization, the pressure upper limits at the overflow port, column section, and large cone section have increased, and the pressure inside the overflow pipe has also risen.

**Fig. 23:**
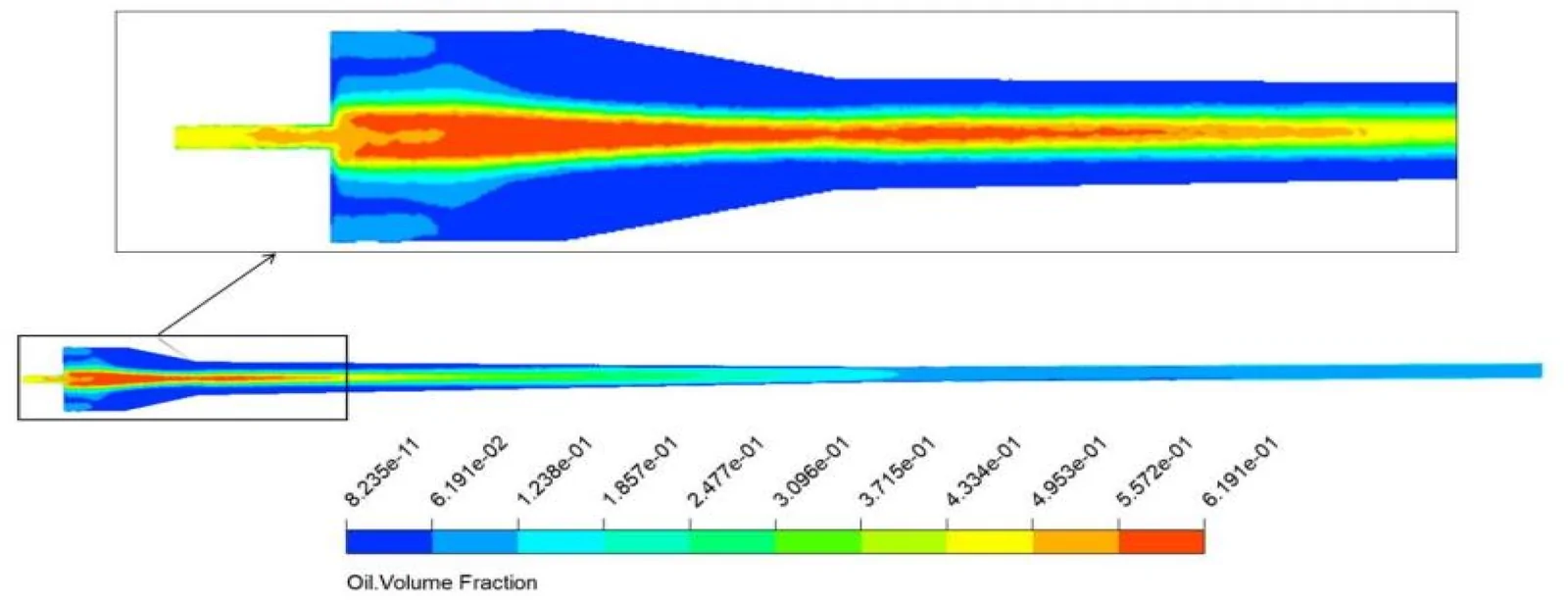
Optimized concentration distribution.

The concentration distribution after optimization of the cyclone tube is shown in Fig. 23.

After optimization, the oil phase concentration distribution shifts upward compared to the pre-optimization oil phase concentration in Fig. 6. Due to the removal of the overflow pipe's insertion, the distance where oil droplets accumulate has been significantly reduced. The optimized cyclone tube shows an overall performance improvement across all parameters compared to the pre-optimization setup.

### 5.4 Engineering applicability, uncertainty, and future perspectives

Although the overall sensitivity ranking indicates that oil droplet diameter, bubble diameter, and inlet flow rate have strong effects on separation performance, these variables are mainly associated with feed properties and operating conditions. For field equipment modification, the most important controllable structural variables are the overflow pipe diameter and the large cone angle. The overflow pipe diameter controls the split ratio. It also determines whether bubble-droplet aggregates concentrated in the central oil core can be effectively discharged through the overflow. The large cone angle affects the radial acceleration gradient. It also regulates the balance between effective centrifugal migration and excessive turbulent shear. Therefore, the increase in separation efficiency from 84.39% to 95.71% is mainly attributed to the coupled adjustment of the overflow pipe diameter and the large cone angle.

Single-factor analysis provides useful baseline trends. However, it cannot fully capture nonlinear interactions among structural parameters and operating conditions. In this study, the L27 orthogonal design and the BP neural network were used to represent these coupled effects. For example, increasing the inlet velocity can enhance centrifugal acceleration. However, an unmatched cone angle may generate excessive shear and destabilize the attached bubble-droplet aggregates. Therefore, the PSO algorithm was used to search the coupled parameter space globally, rather than optimizing isolated variables sequentially.

Several sources of uncertainty should be considered. Spatial discretization uncertainty was controlled through the grid-independence test. The results became stable when the mesh number exceeded 2.2 million cells. Boundary condition uncertainty was reduced by using field-measured inlet oil concentration, droplet size, operating temperature, and treatment flow rate from the H oilfield. Sampling uncertainty in neural network training was reduced by using an orthogonal experimental design rather than random sampling. However, full probabilistic uncertainty quantification was not performed. Methods such as Monte Carlo simulation or polynomial chaos expansion require a large number of three-dimensional multiphase CFD calculations. Their computational cost was beyond the scope of this study.

The present optimization framework uses oil removal efficiency as the primary objective. However, the maximum separation efficiency is not necessarily the most economical operating condition. The optimized configuration, especially the 6.4 mm overflow pipe diameter combined with a strong swirling field, may increase hydraulic resistance and pressure drop. In offshore platform applications, additional pressure drop requires higher pumping power. This may increase energy consumption and operating cost. Therefore, the optimized design should be regarded as a high-efficiency theoretical solution under defined simulation conditions. Future pilot-scale design should introduce multi-objective optimization. Oil removal efficiency, pressure drop, energy consumption, manufacturability, and life-cycle cost should be considered simultaneously.

Several limitations remain. First, bubbles and oil droplets were treated as spherical dispersed phases. Deformation, secondary coalescence, and breakup were not explicitly resolved. Under higher gas injection rates or higher oil concentrations, these processes may alter the local bubble-size distribution and affect separation efficiency. Second, the BP neural network shows strong interpolation ability within the trained parameter range. However, it should not be extrapolated directly beyond the investigated operating domain. Third, the optimized separation efficiency was obtained from a CFD-BP-PSO framework. It still requires physical prototype testing and long-term pilot-scale validation under variable offshore field conditions.

Future work will focus on three aspects. First, population balance modeling will be integrated to describe bubble-size evolution in high-shear swirling flow. Second, the effects of surfactants and demulsifiers will be incorporated into the interfacial interaction model. Third, a multi-objective optimization framework will be developed to consider separation efficiency, pressure drop, energy consumption, and operating cost simultaneously.

## 6 Conclusion

(1) A modified Bloom–Heindel model was developed to describe bubble-droplet collision, attachment, and stabilization-detachment in a high-shear air-flotation cyclone. By incorporating turbulence-induced relative velocity, centrifugal buoyancy, Saffman lift, Magnus force, and a dynamic Bond number, the model links micro-scale interfacial interaction with macro-scale swirling flow behavior. Compared with a conventional model that neglects bubble-droplet interaction and may produce errors of up to 15%, the modified model reduced the average deviation from field measurements to 1.2%.

(2) CFD analysis revealed the flow-field mechanism of air-flotation-enhanced centrifugal separation. Aeration promotes the formation of bubble-oil droplet composite particles, decreases their apparent density, and strengthens centripetal migration toward the overflow region. This mechanism explains the significant improvement in separation efficiency after gas injection under the defined numerical boundary conditions.

(3) The effects of structural and operating parameters were systematically evaluated. Although oil droplet diameter, bubble diameter, and inlet flow rate strongly influence separation performance, the most important controllable structural parameters for field modification are the overflow pipe diameter and the large cone angle. These two parameters jointly determine central oil-core extraction, radial acceleration, and shear stability of bubble-droplet aggregates.

(4) A BP neural-network surrogate model was established using CFD-based orthogonal simulation data. The model achieved a Pearson correlation coefficient greater than 0.99, a test-set MSE of 0.00223785, and more than 95% of prediction errors within ±5%. Based on this surrogate model, PSO optimization identified a field-oriented optimal configuration with a 6.4 mm overflow pipe diameter and a 13° large cone angle, yielding a theoretical maximum separation efficiency of 95.71%.

(5) The present optimization remains a single-objective framework focused on oil removal efficiency. The optimized efficiency should therefore be interpreted as a theoretical prediction rather than a direct field guarantee. Future research should conduct physical prototype and pilot-scale tests, incorporate bubble coalescence and breakup, consider surfactant and chemical additive effects, and develop multi-objective optimization models that balance separation efficiency, pressure drop, energy consumption, and life-cycle cost.

## Conflicts of interest

There are no conflicts to declare.

## Appendices

### Detailed force expressions in the modified bubble–droplet interaction model

In the eqn (4), *i* = *p* represents the oil droplet, and *i* = *b* represents the bubble. *F*_c_, *F*_p_, *F*_d_, *F*_M_, *F*_L_ correspond to the following forces: *F*_c_ is the inertial centrifugal force, *F*_p_ is the centripetal buoyant force, *F*_d_ is the fluid medium resistance, *F*_M_ is the Magnus force, and *F*_L_ is the Saffman force.A1
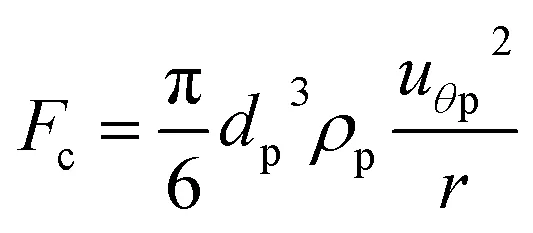
A2
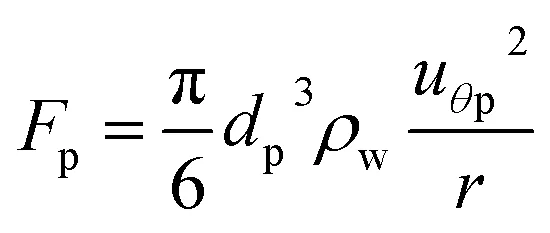
A3
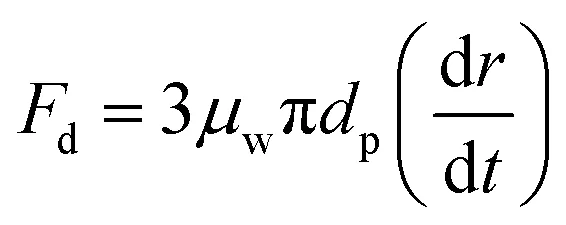

A4 *F*_M_ = *kρ*_w_*d*_p_^3^*ω*(*ν*_*θ*_ − *ν*_*θ*p_)

A5
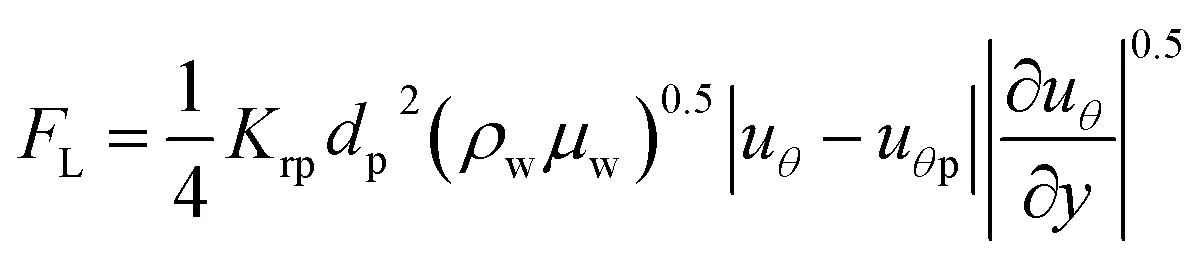
In the equation, *d*_p_ represents the oil droplet diameter (m), *ρ*_p_ is the oil phase density (kg m^−3^), *ρ*_w_ is the water phase density (kg m^−3^), *u*_*θ*p_ is the tangential velocity of the oil phase (m s^−1^), *µ*_w_ is the water phase viscosity (Pa s), and *k*, *K*_rp_ are constants.

In the eqn (10), *F*_ca_, *F*_hyd_, *F*_c_, *F*_p_, *F*_d_, and *F*_σ_ represent the capillary force, hydrostatic pressure, centripetal buoyancy, inertial centrifugal force, mechanical acceleration force, and the force triggered by the bubble interface tension, respectively.

The expressions for the capillary force and hydrostatic pressure, which are the adhesion forces, are as follows:

A6 *F*_ca_ = π*d*_p_*σ* sin(*ω*)sin(*ω* + *θ*)

A7 *F*_hyd_ = 0.25π*d*_p_^2^*ρ*_w_*gh* sin^2^ *ω*

In the above equation,A8
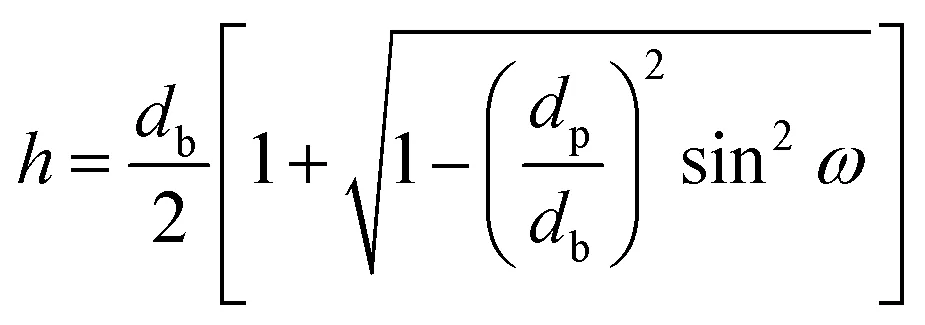
A9
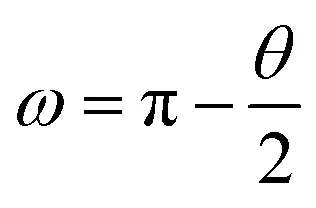


Additionally, the mechanical acceleration force and the force triggered by the bubble interface tension, which are considered detachment forces, can be expressed by eqn (A10) and (A11), respectively.A10
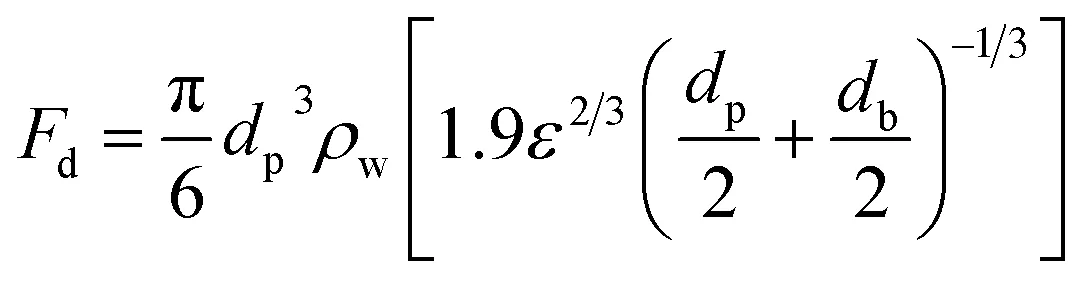
A11
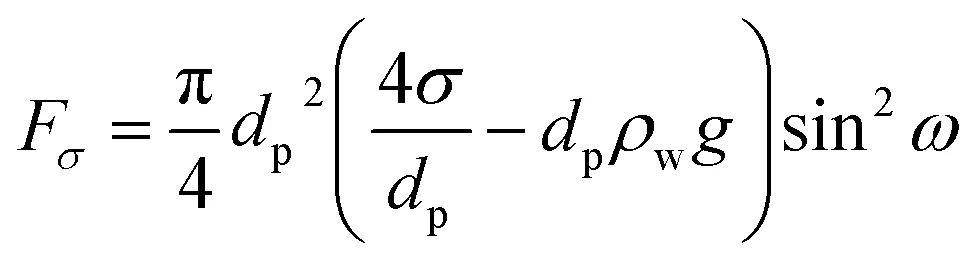


## Data Availability

Data for this article, including the raw simulation dataset and the backpropagation neural network training script, are available at Science Data Bank at https://doi.org/10.57760/sciencedb.46217.
